# Inside the Sickchamber in Early Modern England: The Experience of Illness through Six Objects*

**DOI:** 10.1093/ehr/ceab165

**Published:** 2021-08-16

**Authors:** Hannah Newton

**Affiliations:** University of Reading, UK

A painting by Jacobus Vrel (*c*.1630–80), one of the lesser-known Dutch genre artists, inspired the subject of this article (fig. 1). It depicts a young nurse seated on a low wicker chair, in a bright, sparsely furnished interior; her head rests in her hand after a long night of ‘watching’.^1^

Our eyes are drawn to the contrasting background space, a dark alcove bedchamber, within which lies the shadowy figure of a sick man in bed. The image made me wonder what sickrooms were like in England, a place that lacks a ‘genre tradition’.^2^ Where were these chambers located in English homes, and how did they look, sound, and smell? What sorts of objects might one expect to find in such a room, and how did these material things affect the patient’s experience of illness? Focusing on seventeenth- and early eighteenth-century England, this study transports us imaginatively into the sickchamber, a space which has attracted little historiographical attention, and attempts to reconstruct some of the patient’s sensory and emotional perceptions of this environment.^3^ To do so, a material culture approach is deployed, which involves the analysis of six key objects from the sickroom: a physic vessel, bedcurtains, clock, mattress, sheets, and blankets.

The central argument is that the material environment of the sickchamber substantially shaped the patient’s sensations of sickness, and in turn illness radically altered the way that the sick related to the objects and things around them. When a person was seriously unwell, the sights, sounds, smells, tastes and tactile sensations of the items or spaces which during health usually gave rise to feelings of pleasure and comfort, became sources of emotional and bodily distress. This was because disease was found to ‘assault’ the patient’s sensory powers, the channels through which objects were perceived in early modern thinking. Through investigating these hitherto unexplored effects of illness, the article advances our understanding of the very meaning of disease in early modern England: it shows that illness was conceived as a form of dispossession, the taking away of one’s capacity to appreciate one’s possessions.^4^ A focus on objects also highlights a number of common but rarely examined features of the experience of illness, each of which came to be associated with particular material possessions. These included swallowing a ‘loathsome potion’ (connected to physic vessels), ‘sleep labour’d and disturb’d’ (associated with clocks and mattresses), and ‘sail[ing] thorow all those overflowing sweats’ of fevers (linked to bedclothes).^5^ Ultimately, the article demonstrates that a material approach to medical history enables us to recover aspects of somatic existence which cannot be deciphered from textual sources alone. Such an approach is also particularly suitable for a study of early modern illness, since people at this time believed that bodies and matter were deeply entwined.^6^ As Steven Shapin has observed, everything on earth, from birds to bedsheets, was thought to be composed of the same four elements.^7^ Contemporary epistemological frameworks, in other words, taught that embodiment, and concepts of matter, were inherently environmental. This idea is enjoying a resurgence in ‘new materialist’ studies, with scholars such as Timothy LeCain and Ian Hodder asserting that bodies and minds are ‘inseparable from the material world around us’.^8^ The ensuing discussions lend further support to this view.

At the heart of material culture studies is the idea that objects exert ‘agency’, the capacity to influence human behaviour and elicit emotion. Focusing on the latter power, Sasha Handley and Sherry Turkle refer to possessions as ‘emotional’ or ‘evocative objects’, which play a ‘powerful role … in eliciting, and making visible, a range of entangled affective states across temporal boundaries’.^9^ The present article builds on this work by exploring how such agency operated in practice: it suggests that it was chiefly the sensory qualities of things—or the sensory perceptions to which they gave rise—that evoked an emotional response. The darkness created by drawn bedcurtains, for instance, could elicit feelings of alarm in patients. By placing the senses at the centre of object studies, and drawing explicit connections between emotional and sensory experiences, this study offers a fuller representation of how people from the past actually felt about their material possessions. A discussion of sickness also helps tackle an entrenched challenge in the field of material culture studies: the silence of many historical records on ‘everyday objects’.^10^ Confined to the sickchamber for a stretch of time, the attention of the sick tended to rest on the things around them, and elicited comments in their personal documents which in health would rarely have been voiced. An analysis of these written sources, together with surviving artefacts, is the most fruitful way to discover how the objects were perceived at the time. This combined approach is necessary because, as Pamela Smith has acknowledged, ‘It is really hard to investigate material objects just from the objects themselves’.^11^

Since Roy Porter’s call for a ‘medical history from below’ in 1985, a torrent of scholarship has been produced on the early modern patient.^12^ The first surge of research focused on people’s practical responses to sickness, such as their choices of therapies and practitioners, and the quality of lay medical knowledge.^13^ In the 1990s, historians became increasingly interested in patients’ bodily sensations, as exemplified by Barbara Duden’s seminal text, *Woman Beneath the Skin*.^14^ The burgeoning of the histories of pain and the emotions in the 2000s has significantly enriched this research, with scholars considering the emotional, spiritual and social dimensions of suffering and death.^15^ Finally, in the last two decades, the rise of material, sensory and spatial studies has inspired scholars such as Sandra Cavallo and Tessa Storey to analyse the roles of household objects and lifestyle choices in the preservation of health.^16^ Drawing these various ‘turns’ together, this article zooms in on a chapter of the patient’s story which has rarely been subjected to direct historical analysis: what it was like to be inside the sickchamber.^17^ By combining insights from the interrelated histories of medicine, material culture, emotions, the senses, and spirituality, this study seeks to achieve a more holistic picture of the lived experience of sickness than has previously been possible. The research also adds to recent studies of ‘sensescapes’, the term coined by sensory historians to denote the multisensory and material qualities of particular environments, which so far includes villages, cities, markets, theatres and churches.^18^

The following types of material and textual sources form the basis of this research: a selection of extant objects; diaries, autobiographies and extemporal meditations; personal correspondence; sermons and conduct books; and medical texts, doctors’ casebooks and recipe collections. Surviving artefacts are essential for providing insights into the material and sensory qualities of the sickroom, information which was often ‘blind in plain sight’ in many written sources.^19^ Medical texts and casebooks explain how disease affected the senses, and occasionally describe patients’ reactions to health-related objects.^20^ Other sources of particular value are ‘extemporal meditations’, texts in which ministers and laypeople used everyday sights, sounds and activities to trigger spiritual reflections.^21^ One typical scenario was being ill in bed, which was supposed to give rise to thoughts of Christ’s healing miracles and resurrection. To inspire heartfelt spiritual reflections, these passages are often highly evocative, conjuring up the feelings of the sick as they lay in bed, and frequently referring to the things in the room. Indeed, domestic objects themselves were designed with this in mind: Tara Hamling has demonstrated, for instance, that structures such as fireplaces were supposed to encourage spiritual meditation, as well as to provide physical comfort.^22^

While the above sources may seem diverse in their purposes and characteristics, they exhibit certain commonalities which make their juxtaposition in this study appropriate. Namely, most of the authors or owners embraced the same ‘humoral’ model of the human body, and came from similar socio-economic backgrounds—the middling and upper echelons.^23^ In the case of clergymen diarists and physician authors, there was a degree of occupational sympathy: the care of the body and soul were connected, and ministers were often well versed in medical theory.^24^ Most religious historians now agree that the outlook of puritan clergymen differed from those of other individuals in ‘temperature’ rather than substance; therefore to use spiritual and medical sources in conjunction is reasonable.^25^ It should be noted that while the majority of the objects and texts are English, some of the published medical treatises have European origins. This choice is allowable because the basic therapeutic practices discussed in this article, and their related objects, were shared across Europe, despite some differences between countries regarding other specific aspects of health preservation.^26^

Of course, the above sources are not without their limitations: the main one in this study is the over-representation of the socio-economic elites. Obviously, to read or write required literacy and leisure time, and some of the possessions featured in this study were affordable only to wealthier individuals.^27^ Even the more basic items—such as mattresses—would have varied in quality between different social groups, and it is doubtful whether the poorest in society, who were living in multi-occupied dwellings of few rooms, would have been allocated a separate sickchamber.^28^ The focus of this article therefore necessarily rests on those of middling status and above. The other methodological issue to highlight is the question of whether it is ever possible to access the feelings of people from the past. It is a truism that words do not adequately capture sensory or emotional experience.^29^ Barbara Rosenwein, for example, believes that while we can ‘understand how people articulated … how they felt’, we cannot know how ‘a certain individual [actually] feels in a certain situation’.^30^ Mark Smith has made a similar point in relation to sensory experience, remarking that even if we could recreate (‘produce’) exactly the sounds and other sensations of the past—a task fraught with difficulties—we can never hope to ‘consume’ them in the same way as our ancestors, due to cultural changes.^31^ Nevertheless, I am inclined to take a more optimistic stance, agreeing with Monique Scheer that the divide between the outward expression, and inner experience, of an emotion or sensation has been overstated. She believes that manifestations of feelings—through words or gestures—are inseparable from the emotions themselves.^32^ The same could be said about Smith’s distinction between the ‘production’ and ‘consumption’ of a sensory perception, since what one hears (or sees, smells, and so forth) must influence how one perceives it.^33^ From this position, it *is* possible to gain insights into past feelings: the chosen method is to analyse how contemporaries defined, described and depicted their sensations and emotions, uncovering the historically specific meanings behind particular aspects of illness and their associated objects.

This article focuses on serious physical illnesses that were treated at home. Further, comparative studies will be necessary to establish in what ways the sensescapes of other healing spaces diverged from the domestic sickchamber, such as the hospital, birthing chamber, and apothecary’s shop.^34^ By ‘serious’, I mean diseases which caused considerable bodily suffering, loss of function, or posed a threat to life. This category may seem somewhat amorphous, but in early modern England the common features of illnesses were emphasised much more than they are today. This was partly due to the way disease was defined in Galenic medicine: it denoted impairment in the performance of faculties, caused by the ‘distemperature’ or corruption of the body’s ‘humours’ (constituent fluids), a theory that was applicable to all ailments.^35^ The Church reinforced this unifying approach to illness: conduct books usually deal with sickness in a single chapter, teaching that all illnesses are the fruit of sin, and have in common such things as being ‘confin’d to thy Bed’ and ‘wholly entertain’d with the Extremity of thy pains’.^36^

The timeframe of this study—*c*.1600–1720—witnessed significant developments in the production and distribution of material goods.^37^ With the notable exception of clocks, the items analysed in this study were available throughout the period to those who could afford them, but it is undeniable that the numbers of possessions present in sickchambers must have risen over time.^38^ Even among the poor, Craig Muldrew has shown a significant increase in ownership of household goods during the period, including some of the items examined in this article.^39^ In view of these trends, it can be conjectured that the impact of material culture on the experience of illness must have grown during the period for almost everyone. In the medical sphere, the volume and variety of available drugs expanded over the early modern era, but the basic principles that underpinned treatment—the Hippocratic-Galenic theory of the humours—dominated well into the eighteenth century, despite the rise of competing theories.^40^ With respect to medical treatment, therefore, the picture is one of continuity.

Since the emotions and senses are integral to this study, it may be helpful to explain the meaning of these concepts, and how they were thought to be connected. In Aristotelian thinking, the dominant philosophical tradition in early modern England, the emotions were known as the ‘passions’ of the soul or mind; they were defined as ‘motions’ (physical movements) of the middle part of the triangular soul, the ‘animal’ or ‘sensitive soul’, which were instigated for the preservation of the human.^41^ Like the passions, the ‘external senses’ were functions of the sensitive soul, and were regarded as essential for survival—they enabled living creatures to distinguish between beneficial and pernicious environments.^42^ Influenced by both Aristotle and Galen, most vernacular medical writers accepted that there were five senses—sight, sound, smell, taste and touch—each of which required ‘three thinges’ to work: ‘an Object’ (‘Colours, Sounds, Odors, Sapors and Tactile qualities’), ‘a *Medium*’ (such as light, air or spittle), and ‘an Instrument or Organ’ (the eye, ear, nose, tongue and skin).^43^ Although the precise workings of individual senses differed, the basic process can be summarised as follows: the object emitted its essential quality to the medium, which was then imbibed by special receptacles in the sensory organ (such as ‘teats of the tongue’, tastebuds).^44^ Next, the data entered the nerves at the back of the sensory organ, from where it was carried by special vapours called ‘animal spirits’ to the brain, and collected together in the ‘common sense’, the faculty responsible for processing all sensory information.^45^ At last, this information was passed to the ‘imagination’, which created an overall mental image of the external world, or in our case, the sickchamber; it was this mental picture—’the mind’s eye’—to which the soul responded with its passions.^46^ Owing to the perceived ‘love that is betwixt the body and soul’, the soul usually reacted with joy and delight to sensations which the imagination judged to be sweet or pleasant, while anything bitter or painful to the senses provoked distressing passions, such as grief or disgust.^47^ It is crucial to note, however, that these processes were affected by a person’s state of health: as this article shows, when serious illness struck, the senses became temporarily ‘misaffected’.^48^ Typically, each of the sensory powers was either dulled, heightened or distorted by disease, so that, for instance, soft noises seemed loud, or sweet flavours, bitter, perceptions which in turn brought emotional distress to the soul.^49^ It was these ‘depraved’ sensations that explained why illness altered the way people felt about their material possessions.

After a brief discussion of the location of the sickchamber, the article is structured around the various objects to be examined. The choice of objects is determined by two criteria: the first is the regularity with which the item appears in accounts of sickness. At the height of serious illness, most patients were in bed, which is why the bedstead and its furnishings feature heavily. This would come as no surprise to Angela McShane and Joanne Begiato, who have shown that the bed was ‘literally, economically, spiritually, and … emotionally at the centre of the domestic sphere for much of the early modern period’.^50^ Secondly, the selection of objects is based on their potential to yield a multisensory analysis, which illuminates visual, aural, olfactory, gustatory and tactile aspects. Such an approach responds to recent pleas in sensory studies to investigate several senses together, on the grounds that in practice they do not work in isolation.^51^ It might seem strange to think that an object could be tasted, but in line with current historiographical trends, this article embraces a broad understanding of material culture, which includes liquid consumables such as medicinal potions as well as durable, solid objects, including pewter cups and wooden clocks.^52^ Indeed, the term ‘material object’ is defined here as ‘a thing made or consisting of matter’, a meaning which obviously includes liquids.^53^

## I

Over the course of the sixteenth and seventeenth centuries, room use was becoming increasingly specialised in England, with beds gradually migrating from multipurpose ground-floor ‘halls’ to upstairs chambers, a change facilitated by the emergence of the hearth and improvements in staircase architecture.^54^ Although this development was slow and uneven across society as a whole, the economically privileged families featured in the present study usually did have upstairs bedrooms.^55^ It was within one of these chambers that the sickbed was located. This is evident from the tendency of patients to mention the first time they were able to go *downstairs* after an illness: such a movement was a milestone during recovery, a symbol of returning health.^56^ The rationale behind confining the patient to a bedchamber was to reduce the risk of infection to the rest of the household, while shielding the sick person from the hustle and bustle of the living quarters and street. As the Suffolk medical writer Phillip Barrough (d. 1600) advised in a bestselling medical text, ‘long quiet and rest to the patient’ should be granted, so that ‘Nature’, the body’s internal healing agent, could devote all its energies to the ‘office & work’ of overcoming disease.^57^ The sickchamber was either the patient’s accustomed bedchamber, which in health was likely to have been shared with other relatives or servants, or an unoccupied guest room.^58^

Whether or not patients moved out of their normal chamber would have depended on their status, age, and position within the family. During the dangerous illness of baby Nally Thornton from Yorkshire in 1654, her mother Alice also fell very sick; initially the two lay in adjacent chambers, but the ‘great shriks’ of the ‘poore childe … frighted me [so] extreamely’, wrote Alice, that little Nally was ‘remov[ed] into the Blew Parlor, a great way off [from] me’.^59^ In 1663, Samuel Pepys (1633–1703) recorded that his ‘disorder [of] the blood’ forced him to ‘keep my bed’, where ‘I … slept … alone (my wife lying in the red chamber above)’.^60^

Thus, it was more likely to be those higher up the household hierarchy—parents or husbands—who remained in their own bedchambers during sickness. Lower down the social scale, where space was more restricted, instead of installing the patient in a separate chamber, the communal areas of the household may have been rearranged, with a degree of separation achieved through partitioning off part of the room with a panel, or by ‘framing the bed itself’ as the sickchamber.^61^ Even within homes that were large enough to accommodate a separate sickchamber, there must have been considerable variation in the size and sumptuousness of the room.

## II

Having introduced the sources and the sickchamber, the first of the six objects shall be examined: the vessel of physic. In Hippocratic-Galenic medical theory, most remedies were thought to work by assisting the body’s internal healing agent, Nature, in the expulsion of noxious or surplus humours from the body, the perceived cause of disease.^62^ Numerous types of evacuative treatments were offered, from bloodletting to blisters, but here the focus is on purging potions (laxatives), one of the most common forms of medicine. The key ingredients were herbs of varying degrees of bitterness, from the extremely pungent plants wormwood and aloes to the comparatively mild rhubarb, which were taken infused in liquids like beer or wine.^63^ It was the bitterness of these substances that made them effective: this quality was thought to help evacuate the bad humour by reason of its ‘natural familiarity’ (similarity) to the said humour. Quoting Hippocrates, the Dutch physician Levinus Lemnius (1505–68) explained that ‘Physick[,] when it come[s] into the body, it first … draws unto itself, that which is most … like unto it, then it moves the … humours … and forceth them out’.^64^ This idea, known to laypeople as well as doctors, explains the belief that ‘the medicine must be as bitter as the disease’ for it to work.^65^

**Figure 2:**
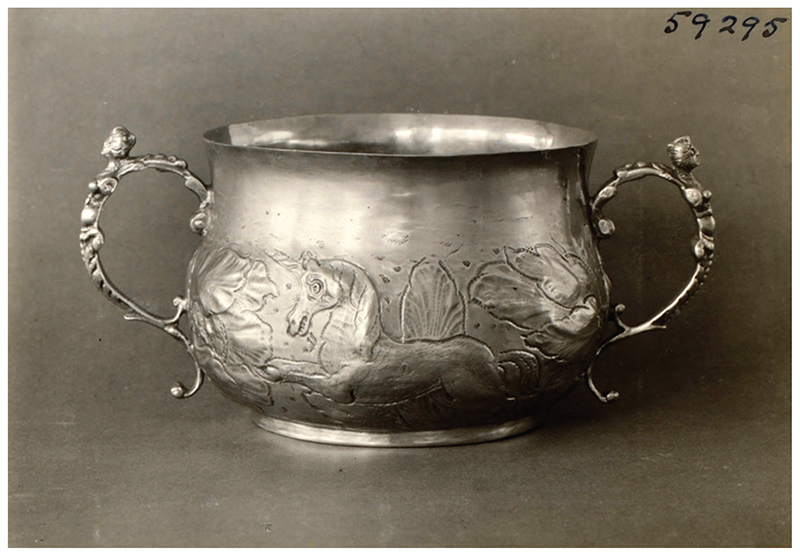
Caudle cup (*c*.1670), unknown maker. © Victoria and Albert Museum, London; museum no. CIRC.980–1925.

It is not easy to establish with certainty what types of vessels were used for the storage and serving of purging medicines.^66^ Only rarely did patients and their families provide this information in their personal documents, perhaps because it was assumed to be too obvious to need stating. One exceptional account can be found in the diary of Elizabeth Walker (1623–92), a puritan minister’s wife: in 1671 she gave her 13-year-old daughter ‘five or six spoonfuls’ of purging potion ‘in a silver cup’, which ‘she received of me without … the least reluctancy’.^67^ Elizabeth offered this unusual detail because she was so amazed at her daughter’s willingness to take the medicine: ‘her stomach did so nauseate’ everything, that she had feared the girl would refuse the purge.^68^ For pious women like Elizabeth, the material composition of the cup may have held spiritual significance: silver was the traditional substance from which the communion chalice was composed.^69^ As such, the sight of this cup—and its feel in the hands and on the lips—may have given rise to meditations on the blood of Christ, and His willingness to heal the sick. A silver cup dating from around the same time is held at the Victoria and Albert Museum (fig. 2). The curators have inferred its medicinal function from its characteristic shape and design: the double handles could easily be gripped by the weak patient, and its sturdy, ‘baluster’ structure meant it would not easily topple over. One side of the cup is embossed with an image of a lion, an animal which Nicole Mennell believes symbolised divine majesty as well as human strength and fierceness.^70^ These associations may have been intended to inspire courage in the face of illness, while reminding the sick to seek God’s blessing before taking a sip. The other side is decorated with an image of a unicorn, a creature often believed to be real in this period, and one which was known for its speed, strength and purity, and for the medicinal potency of its horn.^71^ Such ideas may have helped raise patients’ confidence in the efficacy of the medicine, making them think it would work quickly and powerfully. Decorative silver vessels, however, were hardly likely to have been within the budget of the majority of households, and even among middling groups it is probable that pewter and earthenware were far more common materials. Pewter manufacture flourished across Europe in the seventeenth century, only declining slightly in the early 1700s when crockery began to take over; Craig Muldrew has shown that already by the late 1500s three-quarters of labourers in his inventory sample owned pewter goods.^72^ Less liable to break than earthenware, pewter may have been deemed suitable for the shaky hands of a weak patient. Clues about physic vessels are also provided in manuscript recipe books, wherein writers instruct on medicine dosage and administration.^73^ A remedy containing the laxative senna, for instance, was to be ‘put … into Glass Bottles’ stopped with corks, and taken ‘2 or 3 spoonfulls’ at a time.^74^ Other recipes recommend mixing the potion (or powder) into the patient’s normal drink—usually beer—which suggests that it would have been drunk from an ordinary drinking vessel, such as a tankard.^75^ Medicines might also be given in posset, a milky strengthening drink, and served in a ‘posset cup’ such as that shown in Figure 4, discussed below.^76^

What role did the vessel play in the experience of illness? The visual qualities of these items evoked strong emotional reactions in patients, a finding which demonstrates the agency of objects. William Bullein (*c*.1515–76), a physician and minister from Durham, commented that many patients have such ‘fearful eies’ that ‘it no lesse greveth the[m] to behold or see the vessel, in which the pocion is kept … than to drinke the same … bitter medecine’.^77^ The material composition of the vessel was key—Bullein implied that transparent containers, made of glass, were especially likely to elicit distress in patients, because they could spy the ‘blacknes’ of the liquid.^78^ Other authors suggested that for some patients, the sight of the vessel did not just induce an emotional reaction: it also brought about a physical one, in the form of purging. The expert on melancholy Robert Burton (1577–1640) mentioned a patient cited in the works of Cornelius Agrippa, who, after a ‘memorable and distasteful purge’, was ‘so much moved, *that … the very sight* of the cruze [i.e. container], though he never so much as smelled’ the medicine, ‘would give him a purge; nay, the very remembrance of it would effect it’.^79^ Medical authors attributed this purging power to the affective capacity of body parts, and the sympathy between organs, the imagination, and memory: the sensory organs, stomach and bowels were endowed with an exquisite tenderness or sensitivity, which made them sympathise with, and react to, one another’s sufferings.^80^ As Burton implied, the memory of taking a medicine could be so unpleasant that it permanently altered the patient’s feelings about the vessel. Indeed, this idea was so widespread that it appears in religious writings as a metaphor for expressing spiritual truths. In a discussion of false and true friends, for instance, the London clergyman Thomas Adams (1583–1652) remarked that after taking a ‘lothsome potion’ the patient will ‘ever after hate the very cruze it was brought him in’.^81^

**Figure 3:**
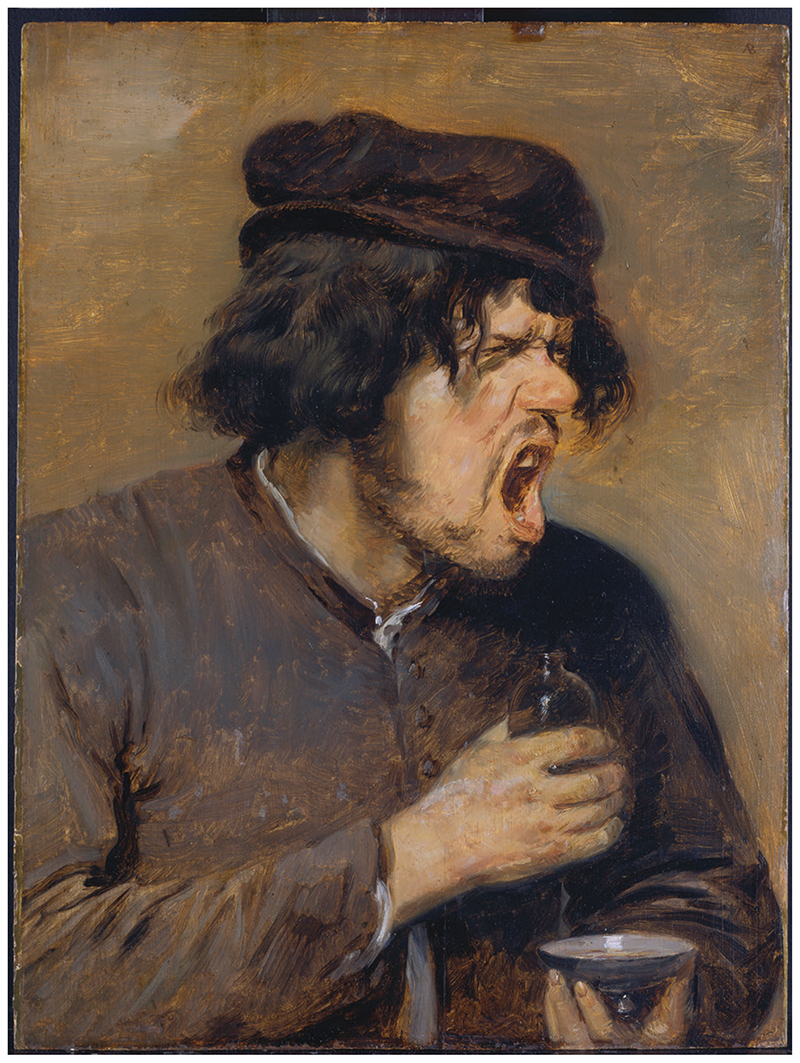
‘The Bitter Potion’ (*c*.1636–8), by Adriaen Brouwer (1605/6–38). Städel Museum, Frankfurt am Main; CC BY-SA 4.0.

Having considered patients’ reactions to the sight of physic vessels, we can turn to their responses to the bitter taste and smell of the medicine itself. The word ‘bitter’ was defined by medical writers as ‘An unpleasant and ungrateful savor … from things taken in … [which] causeth molestation’.^82^ ‘Savor’ (or ‘sapour’) referred both to odour and flavour: smell and taste were thought to work by ‘mutuall consent’—the tongue and nose were located close together, and the sensory data took a similar route to the brain.^83^ The etymology of the word bitter provides clues to the somatic experience of this quality: it comes from the Old English *biter*, which means ‘biting, cutting, sharp’, adjectives associated with acts of violent penetration.^84^ This made sense from a physiological perspective: smells and tastes were often envisaged as material things, which literally passed into the sensory organs. Tastes were ‘watery juices’, which the ‘spongy flesh’ of the tongue was able to absorb, while a scent was a ‘fume, or vapor’, which penetrated the ‘sivelike Bone’ at the back of the nose.^85^ An evocative insight into the imagined reaction of a patient to the gustatory qualities of these drugs is provided in a translation of Boccaccio’s *Decameron*, published in English in 1620: ‘[as] soone as’ the man’s ‘tongue tasted the bitter Aloes, he began to cough and spet extreamly, as being utterly unable to endure the bitternesse’.^86^ Adriaen Brouwer’s painting ‘The Bitter Potion’ (fig. 3) vividly illustrates the patient’s gestural reaction: the man’s face is contorted in an expression of utter disgust—his mouth gapes open, his brows are deeply furrowed, and his eyes are tightly screwed up.^87^ Although this painting is Flemish, English patients responded to bitter medicines in a similar manner. Joseph Hall, for instance, described how he would ‘turne away my head, and make faces, and shut mine eyes; and stop my nostrils, and nauseate, and abhorre to take this … potion’.^88^ Contemporaries explained these gestures by referring to the movement of the ‘vital spirits’, the highly purified vapours which were responsible for carrying out the vital functions of the body and soul: sensations of bitterness made these spirits flee in opposing directions from eyes, nose, mouth and other ‘outward parts’, so that the patient’s whole face appeared distorted.^89^ It was not just the body that reacted to these bitter sapours: owing to the perceived affection between the body and soul, unpleasant tastes and smells also evoked emotional suffering, a tendency evident from the use of the word ‘bitter’ to denote any form of mental distress, especially grief and spiritual guilt.^90^ Indeed, the association between mental suffering and the bitter herbs aloes and wormwood was so well known that these ingredients were deployed as metaphors for all sorts of emotional anguish.^91^

**Figure 4:**
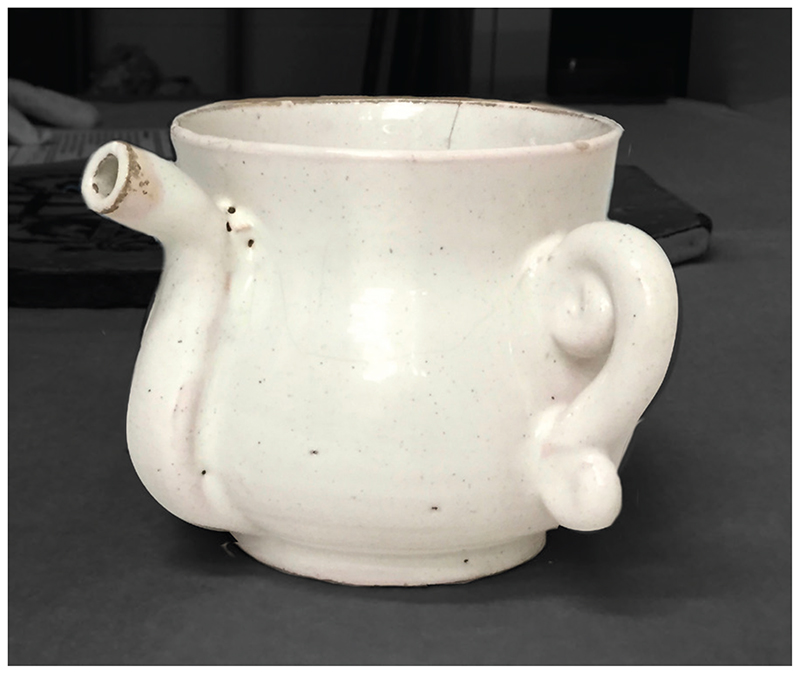
Posset cup (*c*.1670–90). Manchester City Galleries accession no. 1923.327, bequeathed by T.T. Greg. Photograph kindly supplied by Sasha Handley.

So far, this discussion has focused on the sensory experience of physic. The materiality of the vessel itself could influence how a medicine smelled and tasted, as scientists have shown.^92^ For instance, corked bottles and lidded cups would not have emitted much smell until the moment the stopper or cover was removed, in contrast to wide-brimmed, open vessels, from which the scent may have been evident across the room.^93^ Cups with spouts, a design intended to prevent spillage when a medicine was taken in bed, would have limited the volume of liquid entering the mouth, while exposing a smaller proportion of the tongue to the medicine and distancing the drinker’s nose from this substance.^94^ Such properties may have had paradoxical effects, reducing the immediate impact of the unpleasant flavours, while prolonging the time it would have taken to drink the purge. Figure 4, held at Manchester Art Gallery, is an apt example: dating from *c*.1670–90, this globular tin-glazed cup has a long spout that runs from the neck to the base. Although intended primarily for posset, a strengthening drink made from hot milk curdled with ale or wine and flavoured with sugar and spices, it is likely that it was also used for medicines.^95^ We know that flavour is influenced by the shape of the vessel and its connotations—the more rounded the receptacle, the sweeter it seems to taste.^96^ The curved shape of this vessel, together with its association with sweet posset, might have made bitterness less perceptible. Finally, the taste of medicine may have been affected by the vessel’s material composition. Marta Ajmar believes that people in this period were ‘responsive to the smell and the taste of earth’, and certain pieces of pottery were ‘celebrated for the taste that they brought to the food that was cooked in them’.^97^ The same could be said of medical vessels. Pewter cups, for instance, may have given off a sharp taste, owing to the tendency of acidic substances to interact with the lead contained within this material.^98^

It should be noted at this point that bitterness was not always the defining quality of purging potions. Doctors were aware that if the medicine was too disgusting, the patient would be likely to refuse it, or to spit or vomit it up before it had had its desired laxative effect.^99^

To prevent this from happening, it was suggested that the ‘cunning Physician … tempereth his bitter medicines with sweet and pleasant’ ingredients.^100^ By adding sugar or some other sweetener, the drug would remain in the stomach long enough for the medicinal effects to take place. Such an intervention, carried out by lay practitioners as well as physicians, was particularly important when it came to treating children, whose intolerance of bitter tastes was notorious.^101^ However, these adaptations were not always effective: a common complaint from patients of all ages was that the potions continued to taste bitter. Recalling a recent illness, the natural philosopher Robert Boyle (1627–91) observed that some of his remedies had been ‘sweetened with as much Sugar, as if they came not from an Apothecaries Shop, but a Confectioners. But my Mouth is too much out of Taste to rellish anything’.^102^ The Galenic explanation for these altered perceptions was that during acute sickness the tongue is ‘filled with some strange fluid’, which mixes with the gustatory juice of the medicine, so that ‘all things would seem salty to taste, or all bitter’.^103^ So familiar was the experience of altered taste that religious writers found it a useful metaphor to invoke when describing the more abstract idea that sinners fail to relish wholesome counsel. The Yorkshire minister Thomas Watson (d. 1686) wrote in his treatise on repentance, ‘Tis with a sinner, as it is with a sick Patient[:] his pallat is distempered; the sweetest things taste bitter to him: So the word of God which is sweeter than honey-comb, tastes bitter to a sinner’.^104^ In short, the vessel of physic—a ubiquitous object in the sickroom—generated unpleasant multisensory reactions in patients, despite the fact that most individuals recognised that bitterness was the essential quality of an efficacious medicine.^105^

## III

The next object for analysis is the bedcurtain, the ‘visually defining element’ of the early modern bed, and a furnishing which proliferated in English homes during the seventeenth century, even for the very poor.^106^

**Figure 5:**
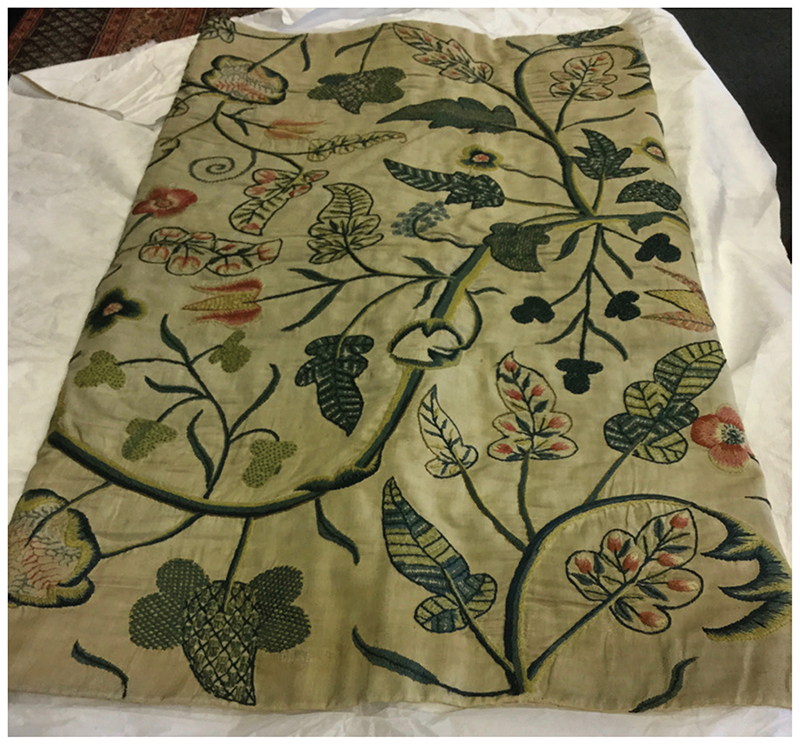
Bed curtain (1680–1730). Manchester City Galleries; accession no. 1986.488. Photograph kindly supplied by Sasha Handley.

**Figure 6:**
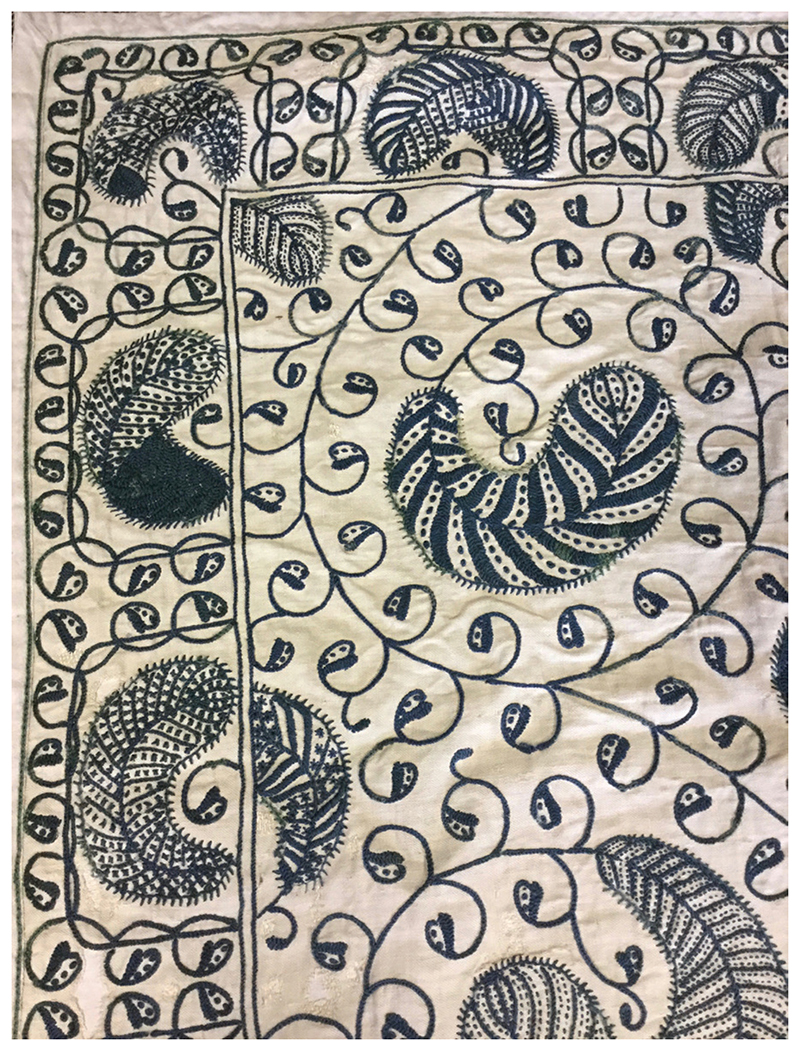
Bed curtain (1660–80). Manchester City Galleries; accession no. 1929.316. Photograph kindly supplied by Sasha Handley.

As far as the sick were concerned, the main function of bedcurtains was to protect the body from the dangerous effects of cold draughts.^107^ Recovery from illness involved the outward movement of bad humours from the interior, ‘noble’ organs to the exterior, less vital parts; warm air helped promote this centrifugal motion by encouraging perspiration from the skin, whereas cold air reversed this process.^108^ The curtains also shielded patients from bright light, something which those in severe pain could not usually ‘abyde’.^109^ David M. Mitchell has shown that, while there was considerable variation in the material composition of bedcurtains between households and across time, when it came to decoration there was a common theme: imagery of the outdoors.^110^ Two rather superior examples can be seen at Manchester Art Gallery: the first (fig. 5) is a pretty crewel-work drape, dating from 1680–1730, and brightly coloured with exotic pink tulips and leaves, a design known as the ‘tree of life’. The other example (fig. 6), from 1660–80, is a white twill-linen hanging, embroidered with blue-green wool to look like the curling feathers of birds. It is possible that such designs were created partly in the interest of health: garden scenes provided patients with an imaginative escape to the outdoors, which in turn was thought to exert a healing effect on the body. Carole Rawcliffe has shown that the ‘delectable sightes and fragrant smelles’ of the outdoors were regarded as therapeutic in premodern England: these sensory delights could help invigorate the spirits, the instruments through which the body’s healer, Nature, operated.^111^ The colour green, so prominent in garden imagery, was deemed especially beneficial.^112^ Owing to the perceived power of the ‘phantasy’ over the body, it was thought to be possible for patients to reap these health benefits simply by imagining the associated sensations. In the case of Figures 5 and 6 these would have included the sweet scent of tulips, and the melodic singing and soft feathers of birds.^113^ While we might think that embroidered imagery would have been the preserve of the wealthy, Sasha Handley has shown that in fact it was possible to ‘personalise’ one’s curtains without great expense.^114^

What was it like lying behind the curtains? The impression conveyed in the personal documents is that it could be a dreary experience, giving rise to feelings of boredom, or, to use the contemporary term, ‘tedium’.^115^ In 1711, the North Yorkshire coal trader Henry Liddell (*c*.1673–1717) complained to a friend, ‘Methinks the time of my confinem[ent] very tedious … which is now near 5 weeks and may be as much longer’.^116^ It was partly the lack of ocular stimulation that made bed so dull—encircled by curtains, there was little to see beyond the surrounding drapes. It might seem strange that patients complained of visual blandness—as was highlighted above, bedcurtains could be highly decorative. However, when the curtains were closed, the bedstead was probably quite dark, even in the daytime, so it is unlikely that the sick would have been able to see the decoration very clearly.^117^ The disease itself could accentuate this lack of visual acuity. The Swiss physician Felix Platter (1536–1614) reported that patients suffering from smallpox and other acute diseases often complained of ‘suffusions’—the appearance of ‘Flies, Smoak, Cobwebs, Filaments, and such like, before [their] Eyes’. He also noted that those afflicted with diseases caused by hot or dry humours, such as convulsions or ‘burning Feavers’, might experience temporary blindness, due to the desiccation of the optic nerve.^118^ Even if the eyesight was not affected in these ways, it was only a matter of time before the sight of the curtains became monotonous. In the words of Charles Cotton (1630–87), ‘Around the place they cast their mournful eyes … And spy no Scenes, but those of yesterday. The same … objects still salute their eye’.^119^ In any case, the suffering of sickness tended to obliterate the sensory pleasure that might otherwise have been felt when looking at beautiful curtains. This is neatly encapsulated in one of Henry Peacham’s (b. 1578) epigrams: he lamented that the presence of worldly comforts to a ‘Soule-sick’ mind will ‘Confere’ the soul ‘no more unto it’s ease … Then do rich curtaines and a Canopie With pearle and gold embroyder’d all about’ to a man ‘who lies [sick] upon the gout’.^120^

Further insights into the experiences of life inside the curtained bedstead are provided in spiritual handbooks and extemporal meditations. The Anglican bishop Jeremy Taylor (*c*.1613–67), described the scene as ‘dressed with darknesse and sorrow’, the patient’s eyes ‘dim as a sullied mirror’ for want of light.^121^ Although darkness may have been easier on patients’ eyes than light, the former carried a burden of its own. Erin Sullivan has shown in her study of sadness that this emotion was closely associated with the humour melancholy, a jet black substance.^122^ Such a link was reinforced by Christian teachings, which invariably associated dark spaces with the frightening concepts of death, hell and sin.^123^ Hell was envisaged as a place of ‘*Utter darkness*’, into which the reprobate were cast: it was this pitch blackness, wrote the Presbyterian Christopher Love (1618–51), which is most ‘apt … to make the hearts of men to tremble’.^124^ No doubt the darkness of the curtained bed actively fostered fears of hell in patients, since the contemplation of damnation was a key duty during illness, part of the religious preparation for death.^125^ This was the case for the parliamentarian army officer William Waller (*c*.1598–1668): when he awoke ‘in a dark Night’, he recalled, ‘all things about me … [were] swadled up in bands of thick darkness’, and he mused that it is not surprising ‘that the most horrid things that we can imagine (*misery, death, hell* it self) are represented by this *black Solitude*’.^126^ Waller uses a material metaphor to describe his experience—he feels ‘swaddled’ up, a reference to the practice of tightly wrapping infants in cloth; the darkness is so impenetrable that he imagines the curtains to be enveloping his whole body and face.^127^ An even more common metaphor invoked in this context referred to prison, a place obviously associated with physical constraint as well as darkness: the bedstead was the cell, and the curtains, the walls.^128^ The high churchman John Kettlewell (1653–95) went as far as to declare that confinement to the sickbed was a ‘more irksome and afflictive’ experience than prison, since ‘your … Bed … is a narrower Compass than a Prison is’.^129^ The prison metaphor was probably chosen for its religious associations: the Bible makes over one hundred references to imprisonment, some of which conjure up the sense of confinement and gloom felt by the sick: Psalm 107, for instance, describes the prisoner as sitting ‘in utter darkness … suffering in iron chains’.^130^

The environment of the curtained bedstead not only gave rise to religious reflections. It could also spur alarm about more worldly things. Robert Boyle described how, during his violent fever, there was a small gap in his bedcurtains, through which he had been able to discern the ‘Dim light of the Candle’. Suddenly, this light ‘considerably increas’d’, which made him suspect that ‘twas … a Thief’ in the room. He put his ‘Head out of the Bed to see whence … [this] unexpected … light proceeded’, and found, to his relief, that it emanated instead from a fault in the tallow candle, which had caused it to blaze in an ‘irregular way’.^131^ The curtains had acted as a visual obstacle, making Boyle feel vulnerable and defenceless; perhaps the fever was partly responsible for his delusion, with the heat affecting his imagination.^132^ In short, life behind the bedcurtains, however ornate, commonly evoked feelings of tedium, and spiritual and temporal unease.

## IV

The next pair of objects for analysis are clocks and mattresses, items which crop up most regularly in accounts of sleeplessness, one of ‘the uneasiest accidents that attend[s] … sickness’.^133^ Doctors attributed insomnia to the corruption or evaporation of a special sleep-inducing vapour in the brain, which in health brought sleep by blocking the nerves.^134^ Patients were more inclined to blame pain.^135^ Among the middling and elite in society, sleeplessness was measured chiefly by the hourly chiming of the clock, a consumer good which proliferated rapidly in the second half of the seventeenth century.^136^ The aforementioned Robert Boyle recalled that during his illness in the 1660s, ‘I … observ’d how long time seem’d to intervene betwixt … the striking of the Clock’, and exclaimed, ‘I think I miss’d not hearing one stroke of the Clock all the Night long’.^137^ This strange impression of time, which David Woods describes as ‘the languorous way in which time itself can seem to unfold’, was commonly observed during periods of physical or mental anguish, cropping up in poetry, plays and prose.^138^ For Boyle, so ‘extraordinar[il]y long’ did the time seem between the chimes that he began to suspect that the instrument, like its owner, must be ‘ill-going’. He ‘call’d out’ to his nurse to fetch him ‘the Watch I use to measure the time’ in his scientific experiments, and found his clock to be an hour slow.^139^ This is an apt example of the reciprocal relationship between illness and objects: sickness undermined this man’s trust in his clock, and the object itself seemed to embody the patient’s sickness. The accuracy of timekeeping had improved by the mid-seventeenth century, thanks to the introduction of the pendulum, which replaced the old balance wheel escapement, a mechanism that had only allowed quarter-hour accuracy; the slowness of Boyle’s clock must therefore have been all the more notable to its owner.^140^ As well as altering patients’ attitudes to their clocks, illness seemed to affect their aural and emotional experience of these instruments. In times of health, the sounds emitted by clocks were usually described favourably. John Smith (*fl*. 1673–80) noted in his handbook on *The Right Managing of Clocks* that when the ‘hammer strike[s] … the Bell … its sound is clear and ful[,] without intermixture of harshness and Jurgelling’.^141^ As signifiers of wealth and status, these ‘emotional objects’—like other luxury goods—commonly evoked feelings of satisfaction and pride.^142^ During sickness, however, the association between clocks and sleeplessness, together with the physical effects of acute illness on the sense of hearing, may have rendered these sounds unpleasant—the chimes seeming muffled rather than clear, harsh rather than mellow. During fever, for instance, it was common for patients to suffer from what Platter called ‘internal sound preternaturally raised in the ears’ (tinnitus), which ‘mixes itself’ with external sounds. Tinnitus was often said to sound like the ‘shrill’ noise which is heard when a ‘little Bell’ is struck; the resulting cacophony of internal and external chimes must have been ‘no smal trouble to a man’.^143^ Such experiences were no doubt exacerbated by the more general tendency of acute illnesses—and especially those accompanied by headaches—to render the patient intolerant of noise of any kind. The Genevan physician Théophile Bonet (1620–89) noted in his casebook that one of his patients was unable to ‘endure the least noise’: the slightest sound made him think ‘a Knife was run into his Brain’.^144^ This metaphor is indistinguishable from the types invoked in descriptions of physical pain, a finding which sheds fresh light on the relationship between pain and the senses in early modern perceptions.^145^ Whereas in modern western medicine pain is regarded as a sense in its own right—’nociception’—Galenic doctors saw it as ‘inherent to all the [five] senses’, and judged any type of sensory discomfort as falling into this category, including irritating sounds like chiming clocks.^146^

**Figure 7:**
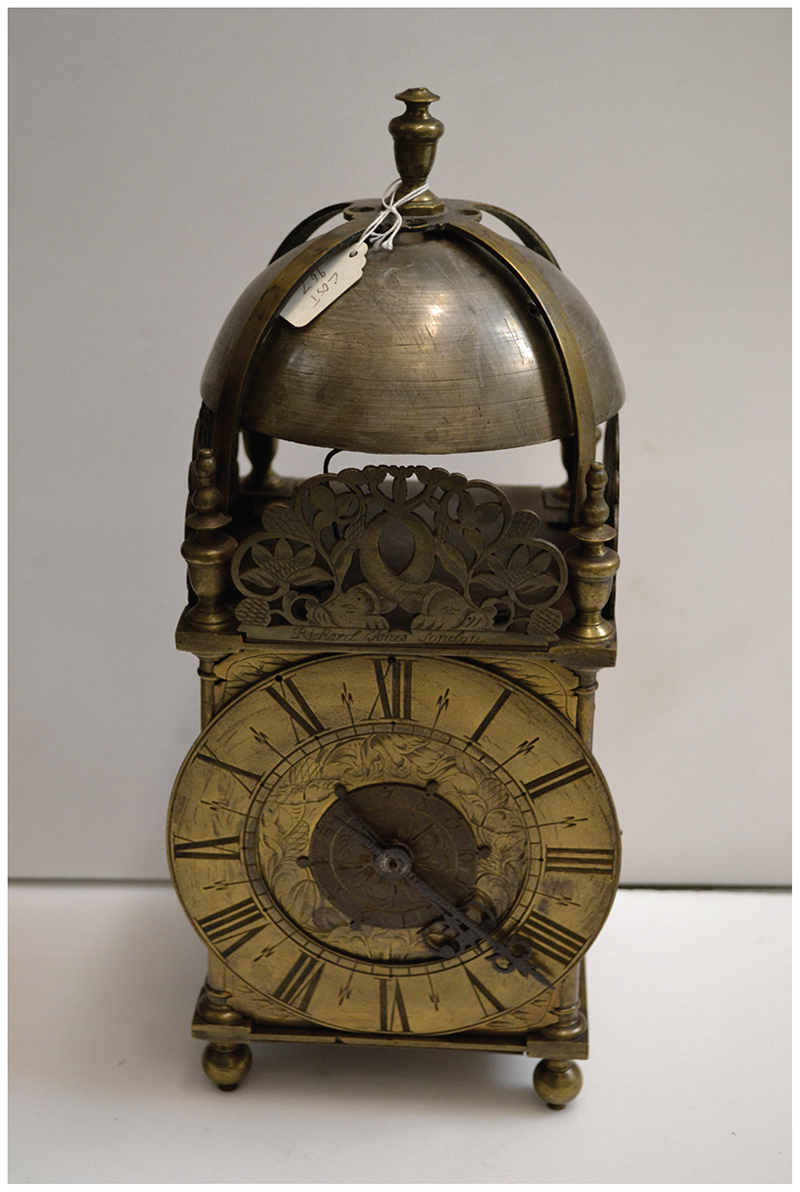
Lantern clock (1650–60), by Richard Ames in London. © Victoria and Albert Museum, London; museum no. M.49–1995, bequeathed by E. Canziani.

Unlike the previous objects examined in this study, it is probable that the clocks that produced the sounds described above were not located in the sickchamber itself, but in other rooms: they were unlikely to have been kept in bedchambers, because they would have awoken the sleeper. This inference is supported by studies of inventories, which show that throughout the early modern period these items tended to be listed in descriptions of halls and parlours rather than in upstairs chambers.^147^ In the 1680s, a new type of clock was developed, the ‘Night Clock’, which was much more suitable for the bedchamber: it contained a lamp, and so enabled the sleeper to tell the time without a candle or chime. An example can be seen in the collections of the British Museum; the numerals are cut out of brass, which allows light from an oil lamp or candle to shine through.^148^ These items were rare, however, perhaps because they were a fire risk. A far more common type of clock in English homes was the ‘lantern clock’, a timepiece invented around the beginning of the seventeenth century, and widely available from the mid-1600s. Figure 7 is a typical example: made in London in 1650–60, this timepiece is made of brass, and is designed to be hung on the wall, or to stand on a bracket. The large bell would have been audible throughout the house, so that even if the clock was not in the sickchamber, the wakeful patient would have been able to hear the chimes, along with the winding sound of the ringing mechanism.^149^ Insights into how these clocks may have sounded are provided in the BBC’s Sound Effects database, though we must remember we lack the ‘period ear’—as well as the state of sickness—to hear the chimes as early modern patients would have done.^150^ Lorna Weatherall has commented that while clocks were present in homes of varying degrees of wealth, people from the lowest socio-economic groups would not have been able to afford these items.^151^ For poor patients, the passing time was probably measured by changing light patterns, together with outside sounds, such as roosters crowing, cows mooing and church bells ringing.^152^

The other object which recurs in descriptions of sleeplessness is the mattress, known in the early modern period as the ‘bed’. Sasha Handley has shown that in this period it was fairly common in wealthy households to stack up to three mattresses on one bedstead: the bottom mattress was usually made of hard-wearing material like straw, to give the sleeper a firm base; the middle mattress would then be composed of wool or horsehair, substances known for their warmth; and the top mattress was stuffed with a softer, cooler, and more expensive material, such as linen, cotton ticking, or feathers.^153^ Although designed for comfort, mattresses were usually found to be uncomfortable during serious illness, especially when the patient was unable to sleep. A physician from Kent, Everard Maynwaringe (b. 1627/28), observed:

*Health*, is *that* which makes your *bed easie*, and your *sleep refreshing …* The *want* of *health* makes … sleep that was [once] stretch[ed] out, from *evening* to the fair *bright day …* broken into pieces, and *subdivided* … the *night* that before seemed *short* is now too *long*; and the downy bed presseth hard against the bones.^154^

**Figure 8:**
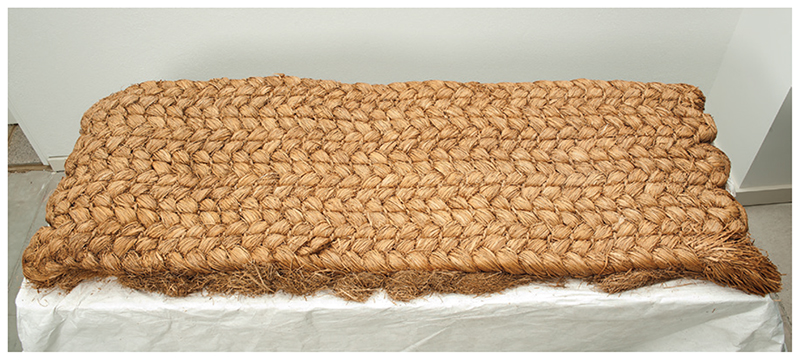
Carex mattress from Titchfield, Hampshire (1600s). Museum of English Rural Life (MERL), University of Reading, object no. 61/242.

As with clocks, the patient’s prolonged contact with the mattress during the wakeful hours of the night seemed to make time move more slowly, an impression which Maynwaringe linked to the effects of sickness on the sense of touch. Many acute illnesses left the skin highly sensitive, a state today known as allodynia; this was what made the soft mattress seem hard. In the case of chronic disease, patients often became emaciated, and it is likely that some may have developed pressure ulcers; the resulting protuberance of the bones greatly contributed to the apparent hardness of the bed.^155^ This was the case for 12-year-old Caleb Vernon, sick of a long consumptive disease in 1665: when his father asked him ‘how he did’, he replied that his ‘*Bones were sore*’; his thigh measured ‘not full four inches about’, and his bones were ‘so sharp as if they would pierce his skin’.^156^ These sharp sensations may also have been the result of a change of mattress: when patients were vomiting, sweating and undergoing other evacuative symptoms or medical treatments, it is likely that the top feather bed was temporarily removed, and replaced with one stuffed with more absorbent, coarser materials, such as seaweed, sedge, or chaff, which could withstand the emission of bodily fluids.^157^ A contemporary proverb states that ‘A downe bed is soft to lye on, but yet it soakes the bodie’.^158^ Figure 8 shows one such replacement mattress, which has fortuitously survived to the present—it was found boarded up in a loft in a house in Titchfield, Hampshire.^159^ The top layer of sedge is twisted into large plaits, so as to help maintain the bed’s structure when wet; this distinctive form must have felt lumpy and hard to lie on. In the case of infectious diseases, these mattresses were supposed to be destroyed after use, since they were deemed to be a source of infection—saturated with the putrid humours that had caused the disease, they were perceived as sick, rather like Boyle’s ‘ill-going’ clock.^160^ Clearly, this mattress was never used during illness, which is why it has survived; it instead functioned as loft insulation.^161^ Of course, lower down the social hierarchy, beds may always have been uncomfortable: rather than sleeping on feather mattresses, servants and labourers were more likely to have rested on beds made from compacted straw, at least at the beginning of our period.^162^

## V

**Figure 9:**
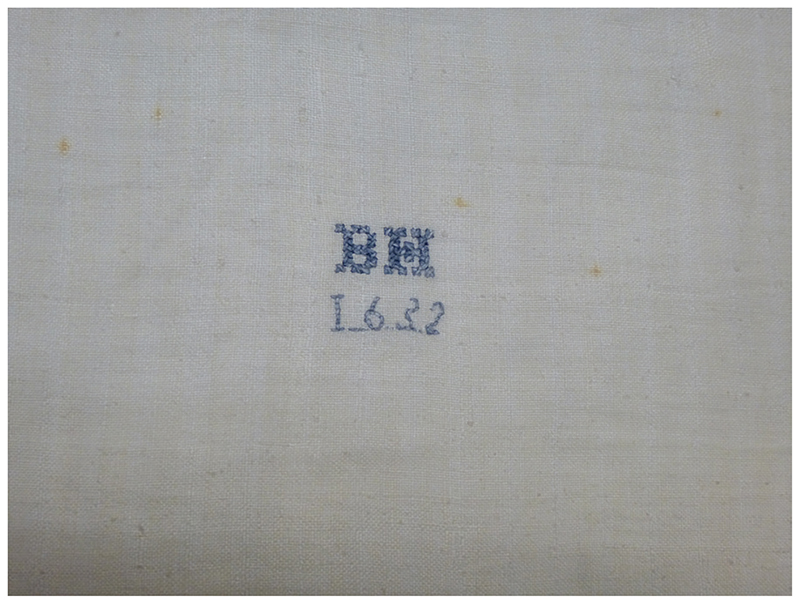
Sheet (1600–1632). © Victoria and Albert Museum, London, museum no. T.54–1958.

The final objects for analysis are bedsheets and blankets, items which can be referred to collectively as the bedclothes. The quality and composition of bedsheets varied greatly between households—and even between bedchambers—but the favoured material was linen, a textile made from woven flax.^163^ Blankets were usually composed of wool, and placed on top of the sheets; in one inventory sample, the mean number of blankets per bedstead was 1.8, but this figure is likely to have changed with the season.^164^ Figure 9 shows a high-quality linen sheet from *c*.1600–32, with the letters ‘E.B’ and ‘B.H’ embroidered in blue cross-stitch, initials of the owners, perhaps a husband and wife. Sasha Handley has suggested that, as ‘cherished personal objects’, sheets and blankets ‘formed a tactile bridge between the waking and sleeping worlds … eas[ing] people’s journeys into restful slumber’ by evoking pleasant sensations. Linen sheets, for example, were known for their smoothness and connotations of purity, while blankets forged feelings of warmth and security.^165^ As revealed below, serious illness usually obliterated these positive experiences to the extent that the objects themselves could be classed as unwell.

In accounts of sickness, the bedclothes are mentioned most frequently in descriptions of sweating. As was stated earlier, recovery involved the evacuation of excess or malignant humours from the body: during fever, the body’s healing agent, Nature, achieved this expulsion by making the patient perspire.^166^ The Dutch doctor Ysbrand van Diemerbroeck (*c*.1609–74) explained that the physician’s role was to assist Nature by ensuring that the patient was ‘well cover’d with Blankets and other Coverings, and so be provoked to Sweat’.^167^ There was also a visual element to this process. James Primrose (1600–1659), a Paris-educated doctor, observed that when treating diseases characterised by pustules physicians often endeavoured to facilitate the evacuation of humours by covering the sick with ‘red cloaths’. It was thought that there was ‘an affinitie’ between this colour and the blood, and so by draping red blankets over the patient, the blood and other humours would ‘spring … forth into [the surface of] the skin’, where they could be safely expelled via the pustules.^168^

Despite understanding that sweating was the route to recovery, most patients found it an ‘afflicting’ or ‘uneasy’ experience.^169^ Typically, the skin felt clammy and wet against the sheets, qualities in this period classed under the sense of touch, and trickling sensations were perceived as the moisture flowed down the body.^170^ The quantity of evacuated matter could be startling. In 1658, Lady Ann Fanshawe’s husband, Sir Richard, contracted a ‘very ill kind of fever’, which put him into ‘perpetual sweats … so violent that it ran down day and night like water’.^171^ The ‘unmeasurable sweat’ suffered by the Leeds antiquary Ralph Thoresby (1658–1725) was ‘almost inconceivable … insomuch, as not only the sheets …, but blankets and rug, were daily dried by the fire’.^172^ Women patients were less inclined to mention sweating in their diaries than men—it carried masculine connotations of violent exercise or sexual activity—but occasionally their husbands describe it.^173^ The politician Simonds D’Ewes (1602–50) told his mother that his wife, Anne, ‘as she lay in bedd, … very ill’ was in ‘soe extreame a sweate as it turned all shee had on into a muck wett’, a muddy puddle.^174^

Sweating under the bedclothes was a thermal experience, as well as a wet one. A universal complaint of feverish patients was the feeling of excessive heat or cold, qualities once again falling into the realm of touch in this period.^175^ Robert Boyle observed that if you were to enter his sickchamber during his fever, and see him ‘not only cover’d, but almost oppress’d, with [bed]Cloaths, [you] would confidently conclude, that, whether or no I be distress’d by the contrary Quality [i.e. heat] … I cannot at least be troubled with Cold’. ‘But alas!’, he cried, ‘in spight of all that lies upon me, an internal Frost has so diffus’d itself through every Part, that my Teeth chatter, and my whole Body does shake strongly enough to make the Bed itself do so’.^176^ This extract conveys the acoustic, as well as the tactile, experience of temperature, which must have been audible to others in the room.^177^ The cold sweat of fever was usually followed by an equally unbearably hot one. Boyle recalled that, as the heat took over, ‘I threw off the Cloaths much faster than’ they had been laid upon him in the former fit, and ‘I, that could a little before scarce[ly] feel all that had been heap’d on me, could not now support a single Sheet’.^178^ As a wealthy gentleman, Boyle’s bedsheet was probably a ‘holland sheet’ like Figure 9, made from fine linen cloth that originated in the Low Countries, and a material prized for its coolness and lightness.^179^ Sickness reversed these thermal and tactile qualities, making the sheets seem hot and heavy. Clearly, Boyle had servants at his beck and call to fetch or relieve him of bedclothes, and no shortage of these items. Among the poor, where access to these material goods was more limited, the experience may have been rather different.

Having discussed the tactile sensations associated with sweating under the bedclothes, we can turn to those of an olfactory nature. While I have no wish to resurrect the now largely rebuffed notion that premodern society was smelly, it is undeniable that many sickbeds were associated with unpleasant odours.^180^ The physician Hermann van der Heyden (1572–1650) sympathised with the patient who longed to be ‘freed from the filthy Stench, wherein he lyes wrapped up’.^181^ Sweating—along with other bodily emissions—was often described as ‘noisome’, the term used by contemporaries to denote anything unpleasant to the senses, especially smell. In the mid-1640s, John Evelyn (1620–1706) was ‘infinitly afflicted with heat & noysomenesse’ from being ‘kept … warme in bed for 16 dayes’.^182^ As Evelyn implies, the main reason for this great stench was a failure to ‘shift’ (change) the bed linen. It was not laziness or olfactory insensitivity that explained such a seemingly neglectful practice. After all, in normal circumstances bedsheets and nightclothes were heavily laundered, as Susan North has demonstrated.^183^ Rather, during acute sickness many people believed that it was actually ‘extream Dangerous’ to change the patient’s bedclothes.^184^ The aforementioned physician Levinus Lemnius ‘severely commanded’ that the ‘Shirts, Sheets, Coverings, Linnen ought [not] to be changed’ until ‘undoubted signes of health shew themselves’, on the grounds that such shifting would ‘presently indanger … their lives’.^185^ Changing the linen usually involved taking the patient out of bed, an action which it was feared would cause the pores of the skin to close, thereby reversing the outward direction of the bad humours.^186^ In the case of contagious diseases such as plague, there was an additional deterrent: changing the linen might infect the person who undertook this chore, since it involved contact with sheets soaked with the cause of disease—bad humours. Effectively, the sheets and blankets had become ill themselves, so sick in fact that they could not be ‘cured’ by laundering, but instead had to be destroyed.^187^

There was, however, disagreement between medical practitioners around this issue: some authorities claimed that a failure to shift the bedclothes could actually exacerbate the sickness. In her bestselling guide for housewives, Hannah Woolley (1622–*c*.1675) criticised the ‘vulgar error of not suffering the diseased … person to change his linen often’: not only would it ‘offend the noses of Visitants’, but it ‘much discourageth and dejecteth the sick person’.^188^ In turn, this emotional distress would ‘sink’ the patient’s ‘natural spirits’, the vehicles through which the body’s internal healer Nature worked, so that this agent lacked sufficient strength to expel the disease.^189^ A patient’s joyful response to being put into clean bedclothes is described vividly by the French surgeon Ambroise Paré (1510–90) in his printed clinical observations: one Lord Auret was afflicted with a ‘great fever’ following a gunshot wound; for two months he had lain in the same sheets. The ‘putrid vapors’ of his sweat, ‘soaking in the sheets’, brought ‘disdain and loathing’ to the patient, took away sleep and appetite, and ‘cause[d] the spirits to … acquire an ill quality’.^190^ At last, the surgeon ordered ‘a bed to be made … where there were [laid] clean white sheets’, into which the patient was lifted. Lord Auret ‘rejoyced much’ to be ‘taken out of his foul stinking bed’, immediately fell asleep, and began to get better.^191^ The state of the sheets was thus a matter of life and death, a perception which encapsulates the perceived potency of objects at this time.

It should be mentioned that even when the bedclothes were not changed, strenuous efforts would have been made to mitigate the unpleasant odours of the sickroom. The London physician Stephen Bradwell commanded that during acute diseases ‘all the houshold-stuffe’ should be ‘perfumed’ to make the ‘ayre … sweet’.^192^ Holly Dugan and Evelyn Welch have shown that the use of fumigations and perfumes was a common measure during sickness.^193^ Unfortunately, however, the sick probably derived little olfactory relief from such interventions. Speaking of perfumed quilts, bedclothes and head coverings, Théophile Bonet warned that ‘very strong scented things … cannot be endured’ in some diseases: such smells ‘make the Head to ake, and cause a turbulent motion in the Spirits’.^194^ This experience was attributed, once again, to the pernicious effects of disease on the senses: the patient ‘judgeth the Scents of things not such as indeed they are, but falsly … as when [one] thinks those [things] that smel wel, do stink’.^195^

## VI

A description of the sickchamber by the Oxfordshire clergyman Robert Harris (*c*.1581–1658) sums up this article’s main argument. He mused that inside this space, you shall find ‘solitarinesse, sadnesse, light shut out, misery shut in … children weeping, wife sighing; the husband groning’. He continued, ‘those senses & parts’ which bring the healthy man comfort ‘occasion the sick man trouble’: ‘the sight of his cupps, glasses [and] boxes makes him sicke, the smell of his meates[,] [makes him feel] sicke, the taste of his drinkes[,] sicke, the least noyse offends him … his bed tyres him, his chaire troubles him’. Harris concluded, ‘poore man, hee is not well, and therefore nothing is well about him; he is sicke, and so all the world is made of sicknesse to him’.^196^ This vignette draws attention to a previously overlooked dimension of illness: the tendency for sickness to adversely affect a person’s sensory powers, which in turn radically altered their perceptions of the things around them. Serious illness transformed what normally would have been objects of satisfaction—shiny drinking vessels, soft mattresses, decorative curtains, resonant clocks and scented bedclothes—into sources of sensory and emotional distress. Such an experience renders intelligible the proverbial sentiment of the time, ‘He that enjoyes his wealth Must alwaies live in health’.^197^ As well as affecting patients’ sensory powers, sickness was thought to have the capacity to change the objects themselves, rendering them ‘sick’. Mattresses and sheets, for example, became so saturated with excreted body fluids, the perceived cause of illness, that they sometimes needed to be destroyed. Clocks were suspected of being ‘ill-going’, due to the elongated way in which time seemed to pass during illness.

What are the implications of these arguments for the histories of medicine and objects? For the former field, the idea that sickness affected patients’ attitudes to their possessions illuminates the bigger concept of disease in early modern England. It suggests that illness was defined as a dis-possession, since the sick felt they were no longer capable of appreciating their material goods. Such an observation is confirmed by the use of the verb ‘recover’ to denote the restoration of health: this word derives from the Anglo-Norman and Middle French *recuvrer*, which means to repossess.^198^ Thus, when patients got better, they regained not just their physical faculties and ease, but also their enjoyment of their possessions. Recollecting his own recent illness, Harris mused, ‘Sicknesse put me out of possession of all, but with health all is come back againe’.^199^ For many patients, this experience must have been literal as well as metaphorical, since sickness could be an expensive episode, owing to the necessity of taking time off work, and paying physicians’ and nurses’ fees.^200^ A discussion of objects has also brought to light various forms of suffering which have tended to be missed in the historiography of illness experience, such as sleeplessness and fever. These symptoms may not be as dramatic as pain—the topic which *has* attracted considerable attention in the literature—but to people at the time they all contributed significantly to the feeling of illness.^201^ This article has necessarily focused on the socio-economic elites. Given that sickness was fundamentally conditioned by the number and types of objects owned, it is likely that poorer people’s experiences of illness may have differed considerably. It is to be hoped that further research, using sources such as hospital records, might provide some insights into the material experiences of poorer patients.

By demonstrating the reciprocal relationship between objects and illness, this article has a significant bearing on material culture studies. It showcases an essential principle in this field: the idea that things are ‘not just passive social constructs but rather active and dynamic forces’, which shape our emotional and bodily experiences of life.^202^ Objects interacted with, and sometimes accentuated, sensations of illness, such as the sharp taste of physic, ‘noyse in the eares’, and ‘fiery sweats’. In turn, the emotions evoked by these forms of sensory suffering were perceived to play a major role in the patient’s illness, either aiding recovery, as in the case of green curtains and red blankets, or exacerbating disease, as we saw for those patients who became ‘impatient of the Stinking smell’ arising from the sheets.^203^ Essentially, it was the patient’s senses that provided the link between objects and experiences, a point of connection which I hope may foment fruitful dialogue between the histories of material culture, bodies and medicine, and emotions.^204^

While this study has emphasised the distressing aspects of life inside the sickchamber, I wish to end by noting that there could be a spiritual silver lining to the material experience of illness, which serves as a useful reminder of the gulf that exists between early modern and modern ideas about the senses and objects in western culture.^205^ Throughout the period of this study, many people seem to have lived through two spheres—the body and soul—and each part was thought to be equipped with its own set of senses: the disturbance or deprivation of the body’s senses during sickness could serve to enhance the acuity of the spiritual senses, with wonderful soteriological consequences.^206^ Robert Boyle remarked that the darkness of the curtained bedstead makes the patient begin to ‘take some notice of his own [spiritual] Condition[,] and his Eyes, for want of outward Objects, are turn’d inwards’ to inspect the state of his soul; this heightened spiritual perception inspired repentance, and ultimately paved the way to paradise.^207^

## Figures and Tables

**Figure 1:**
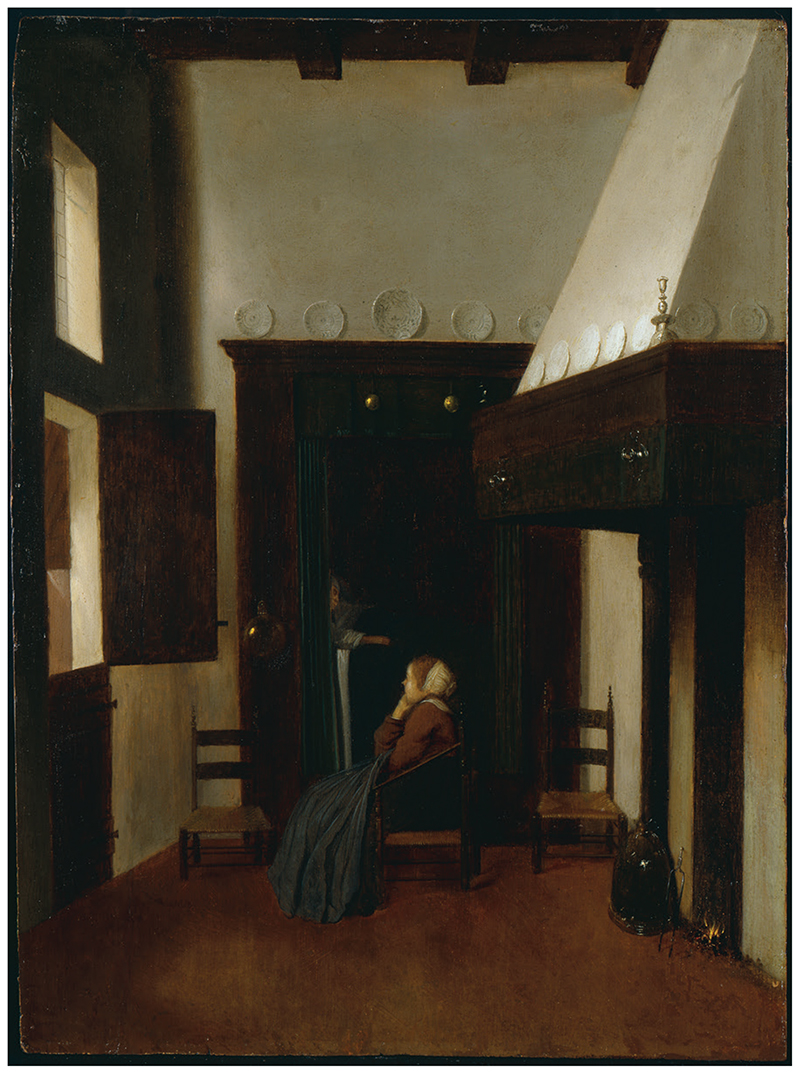
‘The Little Nurse’ (1655), by Jacobus Vrel (*c*.1630–80). © Ashmolean Museum, University of Oxford; accession no. WA1972.287.

## Footnotes

1 Nurses of the sick were known as ‘watchers’ in this period because their chief duty was to keep watch over the patient; on nursing, see M. Pelling, *The Common Lot: Sickness, Medical Occupations, and the Urban Poor in Early Modern England* (Harlow, 1998), ch. 8.

2 Much has been written on this tradition; for example, W. Franits, *Dutch Seventeenth-Century Genre Painting* (New Haven, CT, 2004).

3 See n. 17 below.

4 While historians have not explored the effects of disease on the senses, work has been conducted on the role of the senses in disease diagnosis and treatment. For example: W.F. Bynum and R. Porter, eds., *Medicine and the Five Senses* (Cambridge, 1993); O. Weisser, ‘Boils, Pushes and Wheals: Reading Bumps on the Body in Early Modern England’, *Social History of Medicine*, xxii (2009), pp. 321–39; J. Evans, ‘Female Barrenness, Bodily Access and Aromatic Treatments in Seventeenth-Century England’, *Historical Research*, lxxxvi (2014), pp. 423–43; C. Rawcliffe, ‘“Delectable Sightes and Fragrant Smelles”: Gardens and Health in Late Medieval and Early Modern England’, *Garden History*, xxxvi (2008), pp. 3–21.

5 Thomas Adams, *Diseases of the Soule: A Discourse Diuine, Morall, and Physicall* (London, 1616), p. 70; Joseph Browne, *Institutions in Physick, Collected from the Writings of the Most Eminent Physicians* (London, 1714), pp. 263–5; John Donne, *Devotions vpon Emergent Occasions and Seuerall Steps in My Sicknes* (London, 1624), pp. 599–600. Work has been undertaken on the mechanism of sweating, but few scholars have considered it from patients’ perspectives, with the exception of M. Stolberg, *Experiencing Illness and the Sick Body in Early Modern Europe* (2003; Basingstoke, 2011), pp. 144–5. Sleeplessness has been discussed by several historians, but rarely has it been connected to sickness; see R. Ekirch, *At Day’s Close: A History of Nighttime* (London, 2005), pp. 13–14, 112–13, 288–9.

6 On early modern epistemologies of matter, see P.H. Smith, A.W. Meyers and H.J. Cook, eds., *Ways of Making and Knowing: The Material Culture of Empirical Knowledge* (Chicago, IL, 2014); P.H. Smith, *The Body of the Artisan: Art and Experience in the Scientific Revolution* (Chicago, IL, 2004).

7 S. Shapin, ‘“You Are What You Eat”: Historical Changes in Ideas about Food and Identity’, *Historical Research*, lxxxvii (2014), pp. 377–92, at 379. My thanks to Sasha Handley for this suggestion.

8 Quotation from T.J. LeCain, *The Matter of History: How Things Create the Past* (Cambridge, 2017), pp. 74–5; I. Hodder, *Entangled: An Archaeology of the Relationships between Humans and Things* (Chichester, 2012).

9 Quotation from S. Handley, ‘Objects, Emotions and an Early Modern Bed-Sheet’, *History Workshop Journal*, no. 85 (2018), pp. 169–94, at 188. See also S. Turkle, ed., *Evocative Objects: Things We Think With* (Cambridge, MA, 2011).

10 On the meaning of ‘everyday objects’, see T. Hamling and C. Richardson, eds., *Everyday Objects: Medieval and Early Modern Material Culture and its Meanings* (Farnham, 2010), pp. 12–14. On this challenge, see A. Grassby, ‘Material Culture and Cultural History’, *Journal of Interdisciplinary History*, xxxv (2005), pp. 591–603, at 597–9.

11 ‘Interview with Pamela H. Smith’, *Materialized Identities: Objects, Affects and Effects in Early Modern Culture, 1450–1750* (University of Basel et al., 2016–), available at https://www.materializedidentities.com/single-post/2017/12/01/Interview-with-Pamela-H-Smith (accessed 27 Mar. 2020).

12 R. Porter, ‘The Patient’s View: Doing Medical History from Below’, *Theory and Society*, xiv (1985), pp. 175–98. See also L. McCray Beier’s landmark book, *Sufferers and Healers: The Experience of Illness in Seventeenth-Century England* (London, 1987).

13 For example, R. Porter and D. Porter, *Patient’s Progress: Doctors and Doctoring in Eighteenth-Century England* (Oxford, 1989); D. Nagy, *Popular Medicine in Seventeenth-Century England* (Bowling Green, OH, 1988).

14 B. Duden, *The Woman Beneath the Skin: A Doctor’s Patients in Eighteenth-Century Germany*, tr. T. Dunlap (Cambridge, MA, 1991).

15 For example, see O. Weisser, *Ill Composed: Sickness, Gender, and Belief in Early Modern England* (New Haven, CT, 2015); L. Smith, ‘“An Account of an Unaccountable Distemper”: The Experience of Pain in Early Eighteenth-Century England and France’, *Eighteenth-Century Studies*, xli (2008), pp. 459–80; Stolberg, *Experiencing Illness*; M. Stolberg, *A History of Palliative Care, 1500–1970: Concepts, Practices, and Ethical Challenges* (Würzburg, 2017), ch. 4; R. Anselment, ‘“The Wantt of Health”: An Early Eighteenth-Century Self-Portrait of Sickness’, *Literature of Medicine*, xv (1996), pp. 225–43; H. Newton, *The Sick Child in Early Modern England* (Oxford, 2012), chs. 4, 6; H. Newton, *Misery to Mirth: Recovery from Illness in Early Modern England* (Oxford, 2018), chs. 3, 4, 5. See also the chapters by J.F. van Dijkhuizen, K. Enenkel, M. Schoenfeldt, J. Mayhew, M.A. Lund, and S. Pender, in J.F. van Dijkhuizen and K. Enenkel, eds., *The Sense of Suffering: Constructions of Physical Pain in Early Modern Culture* (Leiden, 2008).

16 S. Cavallo and T. Storey, *Healthy Living in Late Renaissance Italy* (Oxford, 2013); S. Cavallo and T. Storey, eds., *Conserving Health in Early Modern Culture: Bodies and Environments in Italy and England* (Manchester, 2017), pt. 5. See also S. Handley, *Sleep in Early Modern England* (New Haven, CT, 2016).

17 One exception is Mary Ann Lund’s essay, ‘Experiencing Pain in John Donne’s “Devotions Upon Emergent Occasions” (1624)’, in Van Dijkhuizen and Enenkel, eds., *Sense of Suffering*, pp. 323–45, which touches on the sickroom when discussing Donne’s experience of pain. For a nuanced account of the role of the Bible in the sickroom, see R.W. Daniel, ‘“My Sick-bed Covenants”: Scriptural Patterns and Model Piety in the Early Modern Sickchamber’, in E. Clarke and R.W. Daniel, eds., *People and Piety: Protestant Devotional Identities in Early Modern England* (Manchester, 2020). Work which *has* been undertaken on the material environment of the sickroom tends to concentrate on the nineteenth century, the era associated with therapeutic bedrest; see M. Bailin, *The Sickroom in Victorian Fiction: The Art of Being Ill* (Cambridge, 1994); M. Frawley, *Invalidism and Identity in Nineteenth-Century Britain* (Chicago, IL, 2004), ch. 5.

18 See, for example, the essays within the multi-volume series *A Cultural History of the Senses*, gen. ed. C. Classen (6 vols, London, 2014).

19 A. Vickery, *Behind Closed Doors: At Home in Georgian England* (New Haven, CT, 2009), p. 4; L. Weatherill, *Consumer Behaviour and Material Culture in Britain, 1660–1760* (London, 1996), p. 5.

20 On casebooks, see L. Kassell, ‘Casebooks in Early Modern England’, *Bulletin of the History of Medicine*, lxxxviii (2014), pp. 595–625.

21 A. Ryrie, *Being Protestant in Reformation Britain* (Oxford, 2013), p. 112.

22 T. Hamling, ‘Seeing Salvation in the Domestic Hearth in Post-Reformation England’, in J. Willis, ed., *Sin and Salvation in Reformation England* (Farnham, 2015), pp. 223–44, at 224.

23 On this medical model, see n. 62 below.

24 D. Harley, ‘The Theology of Affliction and the Experience of Sickness in the Godly Family, 1650–1714: The Henrys and the Newcomes’, in O.P. Grell and A. Cunningham, eds., *Religio Medici: Medicine and Religion in Seventeenth-Century England* (Aldershot, 1996), pp. 273–92.

25 For example, P. Collinson, *The Elizabethan Puritan Movement* (London, 1967), pp. 26–7; A. Walsham, *Providence in Early Modern England* (Oxford, 1999), p. 2; Ryrie, *Being Protestant*, pp. 6–8.

26 On the differences between Italian and English medical traditions, see Cavallo and Storey, eds., *Conserving Health,* pp. 6–9, 23–52.

27 E. McKay, ‘English Diarists: Gender, Geography and Occupation, 1500–1700’, *History*, xc (2005), pp. 191–212.

28 On poorer people’s domestic goods, see C. Muldrew, *Food, Energy and the Creation of Industriousness: Work and Material Culture in Agrarian England, 1550–1780* (Cambridge, 2011), ch. 4.

29 On the challenges of putting sensations, pain and emotions into words, see M. Jay, ‘In the Realm of the Senses: An Introduction’, *American Historical Review*, xcvi (2011), pp. 307–15, at 309; C. Classen, D. Howes and A. Synnott, *Aroma: The Cultural History of Smell* (London, 1994), p. 3; G. Richards, ‘Emotions into Words—or Words into Emotions?’, in P. Gouk and H. Hills, eds., *Representing Emotions: New Connections in the Histories of Art, Music and Medicine* (Aldershot, 2005), pp. 36–49, at 49; E. Scarry, *The Body in Pain: The Making and Unmaking of the World* (Oxford, 1985), p. 16.

30 B. Rosenwein, ‘Problems and Methods in the History of Emotions’, *Passions in Context*, i (2010), pp. 1–33, at 11. See also J. Bourke, *The Story of Pain: From Prayer to Painkillers* (Oxford, 2014), who believes that sensations of pain *are* accessible.

31 M. Smith, ‘Producing Sense, Consuming Sense, Making Sense: Perils and Prospects of Sensory History’, *Journal of Social History*, xl (2007), pp. 841–58. Much has been written on the challenges of recreating past sounds/sensations; see B. Williams, ‘Scholarship, Authenticity, Honesty’, in D. Greer, ed., *Musicology and Sister Disciplines: Past, Present and Future* (Oxford, 1997), pp. 9–18.

32 M. Scheer, ‘Are Emotions a Kind of Practice (and Is That What Makes Them Have a History)? A Bourdieuian Approach to Understanding Emotion’, *History and Theory*, li (2012), pp. 193–220, at 195–6.

33 Smith, ‘Producing Sense’.

34 There is already interest in the sensescape of the modern hospital, but not its early modern counterpart. See, for example, D. Howes and C. Classen, *Ways of Sensing: Understanding the Senses in Society* (Abingdon, 2014), pp. 57–8; J. Reinarz, ‘Learning to Use their Senses: Visitors to Voluntary Hospitals in Eighteenth-Century England’, *Eighteenth-Century Studies*, xxxv (2012), pp. 505–20.

35 Galen, ‘On the Causes of Symptoms I’, ed. and tr. I. Johnston, *Galen on Diseases and Symptoms* (Cambridge, 2006), pp. 203–35, 184. This text was available in Latin in the early modern period, translated from the Greek by Thomas Linacre as *Galeni Pergameni de symptomatum differentijs liber unus; eiusdem de symptomatum causis libri tres* (London, 1524).

36 For example, R. Allestree, *The Art of Patience and Balm of Gilead under all Afflictions* (1684; London, 1694), Section 2, ‘In Time of Sickness’.

37 Weatherill, *Consumer Behaviour*; M. Overton, J. Whittle, D. Dean and A. Hann, *Production and Consumption in English Households, 1600–1750* (London, 2004).

38 The type of domestic clock featured in this study, the ‘lantern clock’, was invented *circa* 1600, but did not become widely available until the mid-seventeenth century: W.F.J. Hana, *English Lantern Clocks* (Poole, 1979), p. 17.

39 Muldrew, *Food*, p. 196.

40 R. French and A. Wear, eds., *The Medical Revolution of the Seventeenth Century* (Cambridge, 1989); P. Wallis, ‘Exotic Drugs and English Medicine: England’s Drug Trade, *c*.1550–*c*.1800’, *Social History of Medicine*, xxv (2012), pp. 1–27; P. Wallis and T. Pirohakul, ‘Medical Revolutions? The Growth of Medicine in England, 1660–1800’, *Journal of Social History*, lxix (2016), pp. 510–31. On the dominance of Hippocratic-Galenic theory, see S. Pilloud and M. Louis-Courvoisier, ‘The Intimate Experience of the Body in the Eighteenth Century: Between Interiority and Exteriority’, *Medical History*, xlvii (2003), pp. 451–72; Weisser, *Ill Composed*, pp. 19–20.

41 Nicholas Coeffeteau, *A Table of Humane Passions, with their Causes and Effects*, tr. Edward Grimeston (London, 1621), p. 2. On the soul’s structure, see T. Dixon, *From Passions to Emotions: The Creation of a Secular Psychological Category* (Cambridge, 2003).

42 Coeffeteau, *A Table of Humane Passions*, pp. 316–17; Robert Burton, *The Anatomy of Melancholy: What It Is. With all the Kindes, Causes, Symptomes, Prognostickes, and Seuerall Cures of it* (Oxford, 1621), p. 33.

43 Helkiah Crooke, *Mikrokosmographia: A Description of the Body of Man* (London, 1615), pp. 127–8, 616; Burton, *Anatomy of Melancholy*, pp. 32–3. There was controversy over the nature of the sensory organs, especially those of touch and smell; see R. Palmer, ‘In Bad Odour: Smell and its Significance in Medicine from Antiquity to the Seventeenth Century’, in Bynum and Porter, eds., *Medicine and the Five Senses*, pp. 61–8.

44 Michael Ettmüller, *Etmullerus Abridg’ d, or, A Compleat System of the Theory and Practice of Physic* (London, 1699), p. 444.

45 In Galen’s writings, the brain is associated principally with the senses, whereas Aristotelian philosophy taught it was the heart; most early modern medical writers sided with Galen. See F. Quiviger, *The Sensory World of Italian Renaissance Art* (London, 2010), pp. 15–17. On the role of nerves or spirits in sensation, see Crooke, *Mikrokosmographia,* pp. 85, 483–4.

46 On the imagination and other ‘internal senses’, see A. Clements, ‘The Senses in Philosophy and Science: Five Conceptions from Heraclitus to Plato’, in J. Toner, ed., *A Cultural History of the Senses in Antiquity* (London, 2014), pp. 115–38, at 135–7.

47 On the body–soul relationship, see Newton, *Misery to Mirth*, pp. 84, 104–6, 111, 128; ead., *Sick Child*, pp. 199–200.

48 Burton, *Anatomy of Melancholy*, p. 35.

49 On the ways in which the senses could be affected by disease, see Felix Platter, *Platerus Golden Practice of Physick* (London, 1664), pp. 50–87.

50 On the necessity of bedrest during serious illness, see Newton, *Misery to Mirth*, p. 195. A. McShane and J. Begiato, ‘Making Beds, Making Households: The Domestic and Emotional Landscape of the Bed in Early Modern England’, unpublished conference paper, originally presented in 2006, and available at https://www.academia.edu/39035802/Making_beds_making_households_the_domestic_and_emotional_landscape_of_the_bed_in_early_modern_England_Draft_no_references (accessed 17 Dec. 2019).

51 On the advantages of a multisensory approach, see M. Jenner, ‘Follow Your Nose? Smell, Smelling, and Their Histories’, *American Historical Review*, cxvi (2011), pp. 335–51, at 349–50.

52 For example, in his *Matter of History*, Tim LeCain defines ‘things’ as ‘the totality of the organisms, substances, and processes that constitutes the material environment’ (p. 73), a definition which encompasses foods, drinks, and medicines. See also Turkle’s edited volume, *Evocative Objects*, which includes edibles and consumables.

53 *Oxford English Dictionary* [hereafter *OED*], s.v. ‘material object’.

54 On the rise of bedchambers, staircases and hearths, see Handley, *Sleep*, ch. 4; Overton et al., *Production and Consumption*, p. 133; Hamling and Richardson, *A Day at Home*, pp. 29–30, 103, 108, 215, 224.

55 McShane and Bailey, ‘Making Beds’. On labourers’ rooms, see Muldrew, Food, pp. 179–81.

56 Newton, *Misery to Mirth*, pp. 201–2.

57 Philip Barrough, *The Methode of Phisicke* (London, 1583), p. 187.

58 On co-sleeping, see S. Handley, ‘Sociable Sleeping in Early Modern England, 1660–1760’, *History*, xcviii (2013), pp. 79–104, at 101–2; L. Gowing, ‘“The Twinkling of a Bedstaff”: Recovering the Social Life of English Beds, 1500–1700’, *Home Cultures*, xi (2014), pp. 275–304.

59 *The Autobiography of Mrs Alice Thornton of East Newton, Co. York,* ed. Charles Jackson, Surtees Society, lxii (1875), p. 92.

60 *The Diary of Samuel Pepys*, IV: *1663*, ed. R. Latham and W. Matthews (London, 1971), p. 39. See also *The Diaries of Lady Anne Clifford*, ed. D.D.H. Clifford (Stroud, 1990), p. 53.

61 On the use of panels, see Overton et al., *Production and Consumption*, p. 122. Sarah Fox points out that in the context of childbirth, the bed itself ‘framed the birth space’; the same can be said of sickness: S. Fox, ‘Reconceptualising the Birth Process in Eighteenth-Century England’ (Univ. of Manchester Ph.D. thesis, 2017), pp. 50–54.

62 The term ‘Hippocratic-Galenic’ refers to the theory developed by the ancient physicians Hippocrates (*c*.460–370 BC) and Galen (AD 129–199/217). Of course, there existed no single version of ‘Hippocratic’ or ‘Galenic’ medicine; rather, there were many different ‘humoral medicines’: see P. Horden and E. Hsu, eds., *The Body in Balance: Humoral Medicines in Practice* (New York, 2013). On the roles of Nature and evacuation and purging in recovery, see Newton, *Misery to Mirth*, ch. 1.

63 A. Stobart, *Household Medicine in Seventeenth-Century England* (London, 2016), pp. 93–4. On the bitterness of wormwood and aloes, see John Jacob Berlu, *The Treasury of Drugs Unlock’ d, or, A Full and True Description of all Sorts of Drugs, and Chymical Preparations, Sold by Druggists* (London, 1690), p. 38; Joseph Blagrave, *Blagrave’s Supplement or Enlargement to Mr Nich. Culpeppers English Physitian* (London, 1674), pp. 29–30.

64 Levinus Lemnius, *The Secret Miracles of Nature* (1559; London, 1658), pp. 261–2.

65 For example, Donne, *Devotions*, p. 243.

66 Little has been written on English physic vessels; on Italian ‘drug jars’, see V. Avery and M. Calaresu, *Feast and Fast: The Art of Food in Europe, 1500–1800* (Cambridge, 2019), pp. 210–12.

67 Anthony Walker, *The Vertuous Wife, or, The Holy Life of Mrs Elizabth Walker* (London, 1694), pp. 104–5.

68 On children’s attitudes to and experiences of medicine, see Newton, *Sick Child*, pp. 71–4, 176–80.

69 A. Spicer, ‘The Material Culture of Early Modern Churches’, in C. Richardson, T. Hamling and D. Gaimster, eds., *The Routledge Handbook of Material Culture in Early Modern Europe* (Abingdon, 2017), pp. 82–97, at 87.

70 My thanks to Nicole Mennell for discussing this with me, and sharing her forthcoming chapter, ‘The Lion King: Shakespeare’s Beastly Sovereigns’, in K. Raber and H. Dugan, eds., *The Routledge Handbook of Shakespeare and Animals* (London, 2020). See also L. Ryken, J. Wilhoit and T. Longman III, eds., *Dictionary of Biblical Imagery* (Nottingham, 1998) [hereafter *DBI*], pp. 514–15.

71 C. Clark, ‘The Wonders of Unicorn Horns: Preventions and Cures for Poisoning’, 6 Dec. 2012, *The Recipes Project: Food, Magic, Art, Science, and Medicine* (OpenEdition Hypotheses, 2012–), available at https://recipes.hypotheses.org/511 (accessed 29 May 2020); W.P. Gerritsen, ‘The Unicorn and the Pharmacists: Early Modern Views on the Presumed Anti-Toxic effects of Unicorn Horn’, *Gewina*, xxx (2007), pp. 1–10; E. Boran, ‘Unicorn Theory in Early Modern Europe’, *Mythical Creatures at the Edward Worth Library* (Edward Worth Library), available at https://mythicalcreatures.edwardworthlibrary.ie/unicorns/theory/ (accessed 29 May 2020).

72 Weatherill, *Consumer Behaviour*, pp. 8, 28, 31, 41, 63, 110–11, 172, 205; Muldrew, *Food*, pp. 194–5.

73 For the most up-to-date study of recipes, see E. Leong, *Recipes and Everyday Knowledge: Medicine, Science, and the Household in Early Modern England* (Chicago, IL, 2019).

74 Washington DC, Folger Shakespeare Library, Folger Manuscript Transcriptions Collection, 126974, v.a.260, ‘Miscellany’, *c*.1613–*c*.1756, fo. 42r. My thanks to Amie Bolissian Mcrae for sharing this recipe with me. More examples can be found at London, Wellcome Library, MS 108, Jane Baber, ‘A Booke of Receipts’, *c*.1625, fos. 19v, 20v; MS 1071, ‘Lady Barrett’s Select Receipts’, *c*.1700, pp. 3, 61, 62, 65, 93.

75 For instance, A.T., *A Rich Store-House, or Treasury for the Diseased* (London, 1596), p. 23; Paul Barbette, *Thesaurus chirurgiae* (London, 1687), p. 359; Théophile Bonet, *A Guide to the Practical Physician* (London, 1686), p. 96; John Hall, *Select Observations on English Bodies, or, Cures both Empericall and Historicall*, tr. James Cooke (London, 1679), p. 71. On the material culture of alcohol consumption, see P. Witherington et al., *Intoxicants and Early Modernity: England, 1580–1740* (University of Sheffield, 2013–), at https://www.intoxicantsproject.org (accessed 28 Mar. 2020).

76 See n. 95 below.

77 William Bullein, *Bulleins Bulwarke of Defence against all Sicknesse, Soarenesse, and Woundes that Doe Dayly Assaulte Mankinde* (London, 1579), p. 8.

78 Ibid.

79 Burton, *Anatomy of Melancholy*, pp. 195–6 (italics original).

80 On the perceived capacity of organs to feel emotion, see G.K. Paster, *Humoring the Body: Emotions and the Shakespearean Stage* (Chicago, IL, 2004).

81 Thomas Adams, *The Happines of the Church, or, A Description of those Spirituall Prerogatiues wherewith Christ hath Endowed her* (London, 1619), p. 388.

82 Platter, *Platerus*, p. 53.

83 Crooke, *Mikrokosmographia*, pp. 86, 617, 620, 1710.

84 *OED*, s.v. ‘bitter’.

85 Burton, *Anatomy of Melancholy*, p. 34; Crooke, *Mikrokosmographia*, p. 531; Platter, *Platerus*, p. 88.

86 Giovanni Boccaccio, *The Decameron, Containing an Hundred Pleasant Novels*, tr. John Florio (London, 1620), p. 72.

87 On disgust, see R. Firth-Godbehere, ‘Naming and Understanding the Opposites of Desire: The Prehistory of Disgust, 1598–1755’ (Queen Mary Univ. of London Ph.D., 2018).

88 Joseph Hall, *Occasionall Meditations* (London, 1631), p. 344.

89 On the mechanisms behind the knitting of the brows and nose in frowns, see J.B., *Pathomyotamia, or, A Dissection of the Significative Muscles of the Affections of the Minde* (London, 1649), p. 147.

90 On body–soul sympathy, see Newton, *Misery to Mirth*, pp. 84, 104–6, 111, 128; ead., *Sick Child*, pp. 199–200.

91 For example, Mark Frank, *LI Sermons Preached by the Reverend Dr Mark Frank* (London, 1672), p. 391; Lancelot Andrewes, *Sacrilege a Snare: A Sermon Preached, ad clerum, in the Vniversity of Cambridg* (London, 1646), p. 18.

92 For example, H. Schifferstein, ‘The Drinking Experience: Cup or Content?’, *Food Quality and Preference*, xx (2009), pp. 268–76.

93 Examples of posset cups with lids can be seen in the *Intoxicants and Early Modernity* database; for instance, see reference D.O.C./16, at https://www.dhi.ac.uk/intoxicants/record.jsp?searchtype=browse&&source=object&object_ID=49 (accessed 3 Apr. 2020).

94 H.M. Lin et al., ‘More than Just a Utensil: The Influence of Drinking Straw Size on Perceived Consumption’, *Marketing Letters*, xxiv (2013), pp. 381–6.

95 Sasha Handley has kindly informed me that in her research on posset cups at the Folger Shakespeare Library she found that medicines were also administered *in* posset. On posset drinks, see V. Jackson, ‘Shakespeare in 100 Objects: Posset Cup’, 1 Aug. 2011, *Explore Shakespeare* (Shakespeare Birthplace Trust), at https://www.shakespeare.org.uk/explore-shakespeare/blogs/shakespeare-100-objects-posset-cup/ (accessed 28 Mar. 2020).

96 C. Spence and G. Van Doorn, ‘Does the Shape of the Drinking Receptacle Influence Taste/ Flavour Perception? A Review’, *Beverages*, iii (2017), pp. 1–12.

97 ‘Interview with Marta Ajmar’, *Materialized Identities*, https://www.materializedidentities.com/single-post/2018/06/13/interview-with-marta-ajmar (accessed 2 Apr. 2020).

98 K.A. Smith, ‘Why the Tomato was Feared in Europe for More than 200 Years’, *Smithsonian Magazine* (June 2013), available at https://www.smithsonianmag.com/arts-culture/why-the-tomato-was-feared-in-europe-for-more-than-200-years-863735/ (accessed 28 Mar. 2020). On lead poisoning, see J.S. Handler et al., ‘Lead Contact and Poisoning in Barbados Slaves: Historical, Chemical, and Biological Evidence’, *Social Science History*, x (1986), pp. 399–425. My thanks to Sasha Handley and Rachel Winchcombe for alerting me to this reading.

99 Crooke, *Mikrokosmographia*, p. 725; James Primrose, *Popular Errours, or, The Errours of the People in Physick*, tr. Robert Wittie (London, 1651), p. 277.

100 William Kempe, *The Education of Children* (London, 1588), sig. D2r–v.

101 See Newton, *Sick Child*, pp. 85–6.

102 Robert Boyle, *Occasional Reflections upon Several Subjects: Whereto is Premis’ d a Discourse about Such Kind of Thoughts* (London, 1665), pp. 206–7.

103 Galen, ‘On the Causes’, ed. Johnston, p. 189. Such ideas were expressed throughout the period of this study; see Timothie Bright, *A Treatise of Melancholie: Containing the Causes Thereof, and Reasons of the Strange Effects it Worketh in Our Minds and Bodies* (London, 1586), pp. 96–7; Crooke, *Mikrokosmographia*, p. 631; Platter, *Platerus*, p. 53; Etmüller, *Etmullerus Abridg’d*, p. 482.

104 Thomas Watson, The Doctrine of Repentance, Useful for these Times (London, 1668). He is drawing on Proverbs 27:7.

105 A. Wear, *Knowledge and Practice in English Medicine, 1550–1680* (Cambridge, 2000), pp. 89, 405–12.

106 On bedcurtains, see Handley, *Sleep*, pp. 44–5, 114–15, 132–4; Hamling and Richardson, *A Day at Home*, p. 243. On poorer people’s ownership of curtains, see Muldrew, *Food*, pp. 173–7, 194, 196.

107 On the therapeutic uses of curtains in Italian medicine, see S. Cavallo, ‘Invisible Beds: Health and the Material Culture of Sleep’, in A. Gerritsen and G. Riello, eds., *Writing Material Culture History* (London, 2014), pp. 143–50.

108 Newton, *Misery to Mirth*, pp. 50–51, 89.

109 Barrough, *Methode of Phisicke*, p. 13.

110 The most expensive drapes were made of silk or broadcloth, in contrast to the cheaper worsteds and cottons found in more modest chambers: D.M. Mitchell, ‘“My Purple Will be Too Sad for that Melancholy Room”: Furnishings for Interiors in London and Paris, 1660–1735’, *Textile History*, xl (2009), pp. 3–28. On outdoor imagery, see also Handley, *Sleep*, pp. 134, 138–9; B. Smith, *The Key of Green: Passion and Perception in Renaissance Culture* (Chicago, IL, 2009), pp. 45–6.

111 Rawcliffe, ‘“Delectable Sightes and Fragrant Smelles”’. On the role of the spirits as Nature’s instruments, see Newton, *Misery to Mirth*, ch. 1.

112 L. Knight, *Reading Green in Early Modern England* (London, 2014). On green furnishings, see C. Richardson, *Domestic Life and Domestic Tragedy in Early Modern England: The Material Life of the Household* (Manchester, 2007), pp. 210–26, 64–103; Handley, *Sleep*, pp. 138–9; Mitchell, ‘“My Purple”‘.

113 Much has been written on the imagination in early modern Europe. In a medical context, see D. Gentilcore, ‘The Fear of Disease and the Disease of Fear’, in W. Naphy and P. Roberts, eds., *Fear in Early Modern Society* (Manchester, 1997), pp. 184–208. On feathers, and their sensory qualities, see U. Rublack and S. Hanß, ‘Protestant Court Culture and the Language of Feathers’, *Materialized Identities*, at https://www.materializedidentities.com/feathers (accessed 11 June 2021).

114 Handley, *Sleep*, p. 133.

115 The word ‘boredom’ was not in widespread use until the mid-1800s, according to the *OED*.

116 *The Letters of Henry Liddell to William Cotesworth*, ed. J.M. Ellis, Surtees Society, cxcvii (Durham, 1987), p. 48. See also *The Diary of Bulstrode Whitelocke, 1605*–*1675*, ed. R. Spalding (Oxford, 1990), pp. 766–7.

117 On the darkness of the curtained bedstead, and the intimacy that this environment invited, see Hamling and Richardson, *A Day at Home*, pp. 247–8.

118 Platter, *Platerus*, pp. 448, 58. See also Lemnius, *Secret Miracles*, p. 247.

119 Charles Cotton, *The Confinement: A Poem, with Annotations* (London, 1679), p. 14. He was referring to confinement in prison, but this observation was also applicable to the sick, a group frequently compared to prisoners, as we will see below.

120 Cited in Hamling and Richardson, *A Day at Home*; Henry Peacham, *Thalia’s Banquet, Furnished with an Hundred and Odde Dishes of Newly Deuised Epigrammes* (London, 1620), epigram 79 (unpaginated).

121 Jeremy Taylor, *The Rule and Exercises of Holy Dying* (London, 1651), p. 72.

122 E. Sullivan, *Beyond Melancholy: Sadness and Selfhood in Renaissance England* (Oxford, 2016), ch. 1.

123 *DBI*, pp. 191–3.

124 Christopher Love, *Hells Terror, or, A Treatise of the Torments of the Damned* (London, 1653), pp. 18–19. On ideas of hell, see P. Marshall, *Beliefs and the Dead in Reformation England* (Oxford, 2002), ch. 5.

125 R. Houlbrooke, *Death, Religion, and the Family in England, 1480–1750* (Oxford, 1998), ch. 3.

126 William Waller, *Divine Meditations upon Several Occasions: With a Dayly Directory* (London, 1680), p. 1.

127 On swaddling, see H. Newton, ‘Birth, Infancy and Childhood’, in S. Toulalan, ed., *Early Modern Bodies* (forthcoming).

128 See also Donne, *Devotions*, p. 43; Thomas Fuller, *Life Out of Death, a Sermon* (London, 1655), p. 11; London, British Library, Additional MS 42,849, letters of the Henry family, fo. 6r.

129 John Kettlewell, *Death Made Comfortable, or, The Way to Dye Well* (London, 1695), p. 38.

130 *DBI*, pp. 112, 657–9; Psalm 107:10, 12.

131 Boyle, *Occasional Reflections*, pp. 219–20.

132 On delusions/hallucinations during fever, see Lemnius, *Secret Miracles*, p. 65.

133 Boyle, *Occasional Reflections*, p. 211.

134 B. Maclehose, ‘Fear, Fantasy and Sleep in Medieval Medicine’, in E. Carrera, ed., *Emotions and Health, 1200–1700* (Leiden, 2013), pp. 67–94, at 83.

135 For example, Bulstrode Whitelocke ‘slept not all night by his torment’ from piles in 1665: *Diary of Bulstrode Whitelocke*, ed. Spalding, p. 690.

136 Weatherill, *Consumer Behaviour*, pp. 25, 28, 40; Overton et al., *Production and Consumption*, pp. 111–12; P. Glennie and N. Thrift, *Shaping the Day: A History of Timekeeping in England and Wales, 1300–1800* (Oxford, 2009), p. 163. On changes in clock dials over the early modern period, see J. Desborough, *The Changing Face of Early Modern Time, 1550–1770* (Basingstoke, 2019).

137 Boyle, *Occasional Reflections*, p. 214.

138 H. Wood, *Time, Narrative and Emotion in Early Modern England* (Farnham, 2009), pp. 1–3. For an example from poetry, see British Library, Additional MS 45718, commonplace book of Elizabeth Freke, 1684–1714, fo. 82v. On time, see also M. Champion, *The Fullness of Time: Temporalities of the Fifteenth-Century Low Countries* (Chicago, IL, 2017).

139 Boyle, *Occasional Reflections*, pp. 214–15.

140 My thanks to Oliver Cooke, horologist at the British Museum, for this information.

141 John Smith, *Horological Dialogues, in Three Parts: Shewing the Nature, Use, and Right Managing of Clocks and Watches* (London, 1675), p. 29.

142 K. Thomas, *The Ends of Life: Roads to Fulfilment in Early Modern England* (Oxford, 2009), pp. 118, 121.

143 Platter, *Platerus*, p. 80.

144 Bonet, *A Guide to the Practical Physician*, p. 71; see also Barrough, *Methode of Phisicke*, p. 12.

145 On metaphors in descriptions of early modern pain, see J. Mayhew, ‘Godly Beds of Pain: Pain in English Protestant Manuals (*ca*.1550–1650)’, in van Dijkhuizen and Enenkel, eds., *Sense of Suffering*, pp. 299–322; Lund, ‘Experiencing Pain’; Newton, *Sick Child*, pp. 193–8; ead., *Misery to Mirth*, pp. 99–101. For more recent eras, see Bourke, *Story of Pain*, ch. 3.

146 Galen, ‘On the Causes’, ed. Johnston, pp. 203–35, at 220–21, and p. 189. For similar definitions in vernacular texts, see Platter, *Platerus*, p. 220; Coeffeteau, *A Table of Humane Passions*, p. 322.

147 Overton et al., *Production and Consumption*, pp. 126–7; Hamling and Richardson, *A Day at Home*, pp. 41–2; Weatherill, *Consumer Behaviour*, pp. 10–11.

148 See O. Cooke, ‘Time to Shine: A 17th-century Night Clock’, 13 June 2017, *The British Museum Blog* (The British Museum 2010–), available at https://blog.britishmuseum.org/time-to-shine-a-17th-century-night-clock/ (accessed 7 Jan. 2019).

149 My thanks to Oliver Cooke for alerting me to the loud dynamics of lantern clocks.

150 *BBC Sound Effects*, available via https://sound-effects.bbcrewind.co.uk, search term ‘lantern clock’ (accessed 4 June 2021). On the problems of recreating past sounds, see n. 31 above.

151 Weatherill, *Consumer Behaviour*, p. 109. See also Overton et al., *Production and Consumption*, p. 112.

152 Glennie and Shrift, *Shaping the Day*, p. 163. On the sound of church bells, though for a later century, see A. Corbin, *Village Bells: Sound and Meaning in the Nineteenth-Century French Countryside* (Columbia, NY, 1998). Outdoor sounds must have varied according to where patients were living, but we should not assume that animal sounds were only heard in the countryside: they were ubiquitous in cities too; see T. Almeroth-Williams, *City of Beasts: How Animals Shaped Georgian London* (Manchester, 2019); E. Fudge, *Quick Cattle and Dying Wishes: People and their Animals in Early Modern England* (Ithaca, NY, 2019).

153 Handley, *Sleep*, pp. 124–5.

154 Everard Maynwaringe, *The Method and Means of Enjoying Health, Vigour, and Long Life* (London, 1683), pp. 25–6.

155 Newton, *Misery to Mirth*, pp. 15, 65, 70, 71, 77, 81, 91, 120–21, 231.

156 John Vernon, *The Compleat Scholler, or, A Relation of the Life, and Latter-end Especially, of Caleb Vernon* (London, 1666), pp. 39, 65–6.

157 This was the case for bedwetting children and birthing women, as Sasha Handley has shown in *Sleep*, ch. 4. Sarah Fox has made a similar observation in relation to childbirth, and also describes other methods that were used to ‘preserve the feather bed from being wetted or spoiled’, which included dressing the bed with an ‘oiled cloth or dressed sheep skin’, in ‘Reconceptualising the Birth Process’, pp. 50–54. Thanks to Sarah Fox for sharing these extracts with me.

158 Nicholas Breton, *Wits Private Wealth: Stored with Choise Commodities to Content the Minde* (London, 1612), sig. D2v.

159 Handley, *Sleep*, p. 58.

160 M. Healy, ‘Anxious and Fatal Contacts: Taming the Contagious Touch’, in E. Harvey, *Sensible Touch: On Touch in Early Modern Culture* (Philadelphia, PA, 2003) pp. 22–38, at 25.

161 Handley, *Sleep*, p. 58.

162 Muldrew shows that ownership of featherbeds among the poor rose in the 1600s: *Food*, pp. 194–6.

163 Ibid., pp. 49–51, 128–9. The highest quality sheets were ‘holland sheets’, made from fine linen that originated in the Low Countries; lower quality sheets were referred to as ‘coarse flaxen’.

164 Ibid., pp. 126, 130.

165 Handley, *Sleep*, pp. 128–9, 147–8. Of course, not all sheets were smooth—Craig Muldrew’s examination of labourers’ probate inventories shows that these items were often described as ‘old course’ sheets: *Food*, pp. 173, 175.

166 Newton, *Misery to Mirth*, pp. 51, 55.

167 Ysbrand van Diemerbroeck, ‘A Treatise of the Smallpox and Measles’, in his *The Anatomy of Human Bodies, Comprehending the Most Modern Discoveries and Curiosities in that Art* (London, 1694), pp. 1–32, at 15–16.

168 Primrose, *Popular Errours*, pp. 192–4. Primrose himself was sceptical of this theory. See also Van Diemerbroeck, ‘A Treatise’, p. 16.

169 Roger North summed up this experience: his ‘deluge of sweat’ was ‘very uneasy, but salutary’: *Notes of Me: The Autobiography of Roger North*, ed. P. Millard (Toronto, ON, 2000), p. 203. In hindsight, patients attributed their recoveries to sweating: see *An Astrological Diary of the Seventeenth Century: Samuel Jeake of Rye*, ed. M. Hunter (Oxford, 1988), p. 212; Richard Baxter, *Reliquiae Baxterianae, or, Mr Richard Baxters Narrative of the Most Memorable Passages of his Life and Times*, ed. Matthew Sylvester (London, 1696), p. 11.

170 For ‘very Wet’ sensations, see Hermann van der Heyden, *Speedy Help for Rich and Poor, or, Certain Physicall Discourses…* (London, 1653), p. 150; for ‘clammy’, see ‘The Diary of Isaac Archer, 1641–1700’, ed. M. Storey, *Two East Anglian Diaries, 1641–1729*, Suffolk Record Society, xxxvi (1994), pp. 41–200, at 159–60; on trickling sensations, see *The Diary of Ralph Thoresby*, ed. Joseph Hunter (2 vols, London, 1830), ii. 391. The qualities associated with touch are listed in Crooke, *Mikrokosmographia*, p. 648. On early modern touch, see J. Moshenska, *Feeling Pleasures: The Sense of Touch in Renaissance England* (Oxford, 2014).

171 *Memoirs of Lady Fanshawe, Written by Herself*, ed. Nicholas Harris Nicolas (London, 1829), pp. 125–6.

172 *Diary of Ralph Thoresby*, ed. Hunter, i. 124; see also *The Life of Mr Robert Blair, Minister of St Andrews: Containing his Autobiography, from 1593 to 1636*, ed. Thomas M’Crie (Wodrow Society; Edinburgh, 1848), pp. 17–18.

173 M. Stolberg, ‘Sweat: Learned Concepts and Popular Perceptions’, in H. King and C. Zittel, eds., *Blood, Sweat and Tears: The Changing Concepts of Physiology from Antiquity into Early Modern Europe* (Leiden, 2012), pp. 503–22, at 503.

174 *The Autobiography and Correspondence of Sir Simonds D’Ewes, Bart*., ed. James Orchard Halliwell (2 vols, London, 1845), ii. 85–7.

175 H. Roodenburg, ed., *A Cultural History of the Senses in the Renaissance* (London, 2014), p. 5.

176 Boyle, *Occasional Reflections*, pp. 192–3. See also *Letters of Henry Liddell*, ed. Ellis, p. 2.

177 On other sounds of sickness, see Newton, *Misery to Mirth*, pp. 115–17.

178 Boyle, *Occasional Reflections*, p. 193.

179 Handley, *Sleep*, p. 130.

180 For a rejection of this smelly notion, see M. Jenner, ‘Civilization and Deodorization? Smell in Early Modern English Culture’, in P. Burke, B. Harrison and P. Slack, eds., *Civil Histories: Essays Presented to Sir Keith Thomas* (Oxford, 2000), pp. 127–44. Revisionist work on early modern hygiene and sanitation has shown that, in fact, early modern societies were deeply wary of stench, and made strenuous efforts to deal with it: see L. Skelton, Sanitation in Urban Britain, 1560–1700 (London, 2016); N. Korda and E. Lowe, ‘In Praise of Clean Linen: Laundering Humours on the Early Modern English Stage’, in Richardson, Hamling and Gaimster, eds., *Routledge Handbook of Material Culture*, pp. 306–21; S. North, *Sweet and Clean? Bodies and Clothes in Early Modern England* (Oxford, 2020).

181 Van der Heyden, *Speedy Help*, p. 150.

182 *The Diary of John Evelyn*, II: *Kalendarium, 1620–1649*, ed. E.S. de Beer (Oxford, 1955), p. 522.

183 From an examination of extant linens, North has shown that these items were designed to be able to withstand rigorous and regular laundering; see *Sweet and Clean?*, esp. chs. 7, 8 and 9.

184 Van der Heyden, *Speedy Help*, p. 152.

185 Lemnius, *Secret Miracles*, pp. 255–7. North discusses the perceived dangers of changing the fever patient’s linen in *Sweet and Clean?*, pp. 60–66.

186 On this physiological process, see Newton, *Misery to Mirth*, pp. 50–51.

187 Stephen Bradwell, *Physick for the Sickness, Commonly Called the Plague* (London, 1636), pp. 15–16.

188 Hannah Woolley, *The Gentlewomans Companion, or, A Guide to the Female Sex* (London, 1673), p. 165; see also Primrose, *Popular Errours*, pp. 137–8.

189 On the spirits, and the vital role of emotions in causing or curing illness, see Weisser, *Ill Composed*, chs. 3, 4; Newton, *Misery to Mirth*, pp. 17, 38, 58, 84–6, 181.

190 Ambroise Paré, *The Workes of that Famous Chirurgion Ambrose Parey*, tr. Thomas Johnson (London, 1665), p. 775.

191 Ibid., p. 776. On restored sleep as a sign of recovery, see Newton, *Misery to Mirth*, pp. 76–8.

192 Bradwell, *Physick for the Sickness*, p. 15.

193 E. Welch, ‘Scented Buttons and Perfumed Gloves: Smelling Things in Renaissance Italy’, in B. Mirabella, ed., *Ornamentalism: The Art of Accessories* (Ann Arbor, MI, 2011), pp. 13–39; H. Dugan, *The Ephemeral History of Perfume: Scent and Sense in Early Modern England* (Baltimore, MD, 2011), ch. 4.

194 Bonet, *A Guide to the Practical Physician*, p. 68.

195 Platter, *Platerus*, p. 87.

196 Robert Harris, *Hezekiahs Recovery, or, A Sermon* (London, 1626), pp. 30–32.

197 Lemnius, *Secret Miracles*, p. 344. See also N.R., *Proverbs English, French, Dutch, Italian, and Spanish* (London, 1659), pp. 49, 61.

198 *OED*, s.v. ‘recover’.

199 Harris, *Hezekiahs Recovery*, p. 31.

200 On the financial experience of sickness, see Weisser, *Ill Composed*, ch. 6; Newton, *Misery to Mirth*, pp. 222–3.

201 On the meaning of the term ‘feeling ill’, see Newton, *Misery to Mirth*, pp. 98–106.

202 LeCain, *Matter of History*, p. 80.

203 Quotation from Van der Heyden, *Speedy Help*, pp. 152–3.

204 On the relationship between the histories of objects, the emotions and the senses, see Gerritsen and Riello, eds., *Writing Material Culture History*, pp. 7, 151–6, 165–72.

205 On the spiritual advantages of sickness, see J.F. van Dijkhuizen, ‘Partakers in Pain: Religious Meanings of Pain in Early Modern England’, in id. and Enenkel, eds., *Sense of Suffering*, pp. 189–220; H. Smith, ‘Metaphor, Cure, and Conversion in Early Modern England’, *Renaissance Quarterly*, lxvii (2014), pp. 473–502; Newton, *Sick Child*, pp. 204–8; ead., *Misery to Mirth*, ch. 4.

206 S. Coakley and P. Gavrilyuk, eds., *The Spiritual Senses: Perceiving God in Western Christianity* (Cambridge, 2012).

207 Boyle, *Occasional Reflections*, p. 212.

